# *Pseudomonas aeruginosa* MipA-MipB envelope proteins act as new sensors of polymyxins

**DOI:** 10.1128/mbio.02211-23

**Published:** 2024-02-12

**Authors:** Manon Janet-Maitre, Viviana Job, Maxime Bour, Mylène Robert-Genthon, Sabine Brugière, Pauline Triponney, David Cobessi, Yohann Couté, Katy Jeannot, Ina Attrée

**Affiliations:** 1 Team Bacterial Pathogenesis and Cellular Responses, University Grenoble Alpes, IBS, UMR5075, Grenoble, France; 2 UMR6249 Chrono-Environnement, UFR Santé, University of Franche-Comté, Besançon, France; 3 French National Reference Center for Antibiotic Resistance, Besançon, France; 4 University Grenoble Alpes, CEA, INSERM, UA13 BGE, CNRS, CEA, FranceGrenoble; 5 University Grenoble Alpes, IBS, UMR5075, Team Synchrotron, Grenoble, France; 6 Department of Bacteriology, Teaching Hospital of Besançon, Besançon, France; University of Pretoria, Pretoria, South Africa

**Keywords:** *Pseudomonas aeruginosa*, antibiotic resistance, polymyxin, ParR/ParS, *arn*, two-component system, signal transduction, MexXY-OprA, proteomics, nano-DSF

## Abstract

Due to the rising incidence of antibiotic-resistant infections, the last-line antibiotics, polymyxins, have resurged in the clinics in parallel with new bacterial strategies of escape. The Gram-negative opportunistic pathogen *Pseudomonas aeruginosa* develops resistance to colistin/polymyxin B by distinct molecular mechanisms, mostly through modification of the lipid A component of the LPS by proteins encoded within the *arnBCDATEF-ugd* (*arn*) operon. In this work, we characterized a polymyxin-induced operon named *mipBA*, present in *P. aeruginosa* strains devoid of the *arn* operon. We showed that *mipBA* is activated by the ParR/ParS two-component regulatory system in response to polymyxins. Structural modeling revealed that MipA folds as an outer-membrane β-barrel, harboring an internal negatively charged channel, able to host a polymyxin molecule, while the lipoprotein MipB adopts a β-lactamase fold with two additional C-terminal domains. Experimental work confirmed that MipA and MipB localize to the bacterial envelope, and they co-purify *in vitro*. Nano differential scanning fluorimetry showed that polymyxins stabilized MipA in a specific and dose-dependent manner. Mass spectrometry-based quantitative proteomics on *P. aeruginosa* membranes demonstrated that ∆*mipBA* synthesized fourfold less MexXY-OprA proteins in response to polymyxin B compared to the wild-type strain. The decrease was a direct consequence of impaired transcriptional activation of the *mex* operon operated by ParR/ParS. We propose MipA/MipB to act as membrane (co)sensors working in concert to activate ParS histidine kinase and help the bacterium to cope with polymyxin-mediated envelope stress through synthesis of the efflux pump, MexXY-OprA.

**IMPORTANCE:**

Due to the emergence of multidrug-resistant isolates, antibiotic options may be limited to polymyxins to eradicate Gram-negative infections. *Pseudomonas aeruginosa*, a leading opportunistic pathogen, has the ability to develop resistance to these cationic lipopeptides by modifying its lipopolysaccharide through proteins encoded within the *arn* operon. Herein, we describe a sub-group of *P. aeruginosa* strains lacking the *arn* operon yet exhibiting adaptability to polymyxins. Exposition to sub-lethal polymyxin concentrations induced the expression and production of two envelope-associated proteins. Among those, MipA, an outer-membrane barrel, is able to specifically bind polymyxins with an affinity in the 10-µM range. Using membrane proteomics and phenotypic assays, we showed that MipA and MipB participate in the adaptive response to polymyxins via ParR/ParS regulatory signaling. We propose a new model wherein the MipA-MipB module functions as a novel polymyxin sensing mechanism.

## INTRODUCTION

*Pseudomonas aeruginosa* is a Gram-negative opportunistic pathogen which thrives in a wide range of environments and displays high intrinsic resistance to antibiotics, the latter being one of the main threats to the modern healthcare system. In 2017, the World Health Organization classified *P. aeruginosa* as a critical priority pathogen for which the development of novel antibiotics is urgently needed. Polymyxins, a class of antibiotics which includes polymyxin B (PMB) and polymyxin E (PME, also known as colistin), are currently used as a last resort to treat multidrug-resistant *P. aeruginosa* infections (1, 2). Polymyxins are amphipathic cationic anti-microbial peptides (cAMPs) which interact with the negatively charged lipid A component of the lipopolysaccharide (LPS), resulting in its destabilization and loss of outer-membrane integrity. Although the exact mechanism of bacterial killing is still elusive, polymyxin insertion in the outer membrane alters membrane curvature and stability (3). In the proposed model, self-promoted uptake of polymyxin leads to a contact between the inner and outer membranes, allowing phospholipid exchange, in turn creating osmotic imbalance, and eventually leading to bacterial cell death (4–6). While polymyxins have a strong bactericidal effect on *P. aeruginosa*, the latter can adapt to polymyxin stress by inducing a set of eight genes, named *arnBCDATEF-ugd* (*arn*). As a final product, Arn enzymes synthesize the 4-amino-4-deoxy-L-arabinose (L-Ara4N) moiety that is transferred to a nascent lipid A in the inner membrane by ArnT (7). This LPS modification reduces the overall negative charge of the outer membrane, decreasing the affinity of polymyxins toward the bacterial surface.

Expression of the *arn* operon is tightly regulated in response to external stimuli by at least four two-component regulatory systems (TCSs) composed of a membrane sensor histidine kinase (HK) and a response regulator (RR) (8). PhoP/PhoQ and PmrA/PmrB are able to activate the *arn* locus in response to low magnesium concentration, whereas ParR/ParS and CprR/CprS respond to the presence of different cAMPs, including polymyxins, through an unknown mechanism (9–11). In addition to the *arn* operon, ParR/ParS system down-regulates the expression of the OprD major porin gene, which contributes to carbapenem entry into *P. aeruginosa*. ParR/ParS also up-regulates the expression of *pmrAB*, *mexXY-oprM/A* operon coding for an efflux pump, and that of the gene *PA1797*, encoding an uncharacterized protein annotated as a putative β-lactamase (9, 12). Amino acid substitutions in either ParR or ParS were associated with a significant decrease of polymyxin susceptibility in clinical strains of *P. aeruginosa* due to overexpression of the *arn* operon, as well as to carbapenems and MexXY-OprM/A efflux substrates (cefepim, fluoroquinolones, and aminoglycosides) (13–15).

Recent studies investigating the genetic diversity of *P. aeruginosa* isolates revealed five distinct phylogenetic groups/clades (16, 17). According to the classification from reference 16, the phylogenetic group 3, harboring the fully sequenced strain PA7 (18), was the most distant to the two predominant groups, represented by reference strains PA14 (group 1) and PAO1 (group 2). Comparison of gene content of different groups revealed that all genes encoding the type 3 secretion system (T3SS) were absent from both groups 3 and 5. These groups encoded a cytolytic two-partner secretion system, ExlB/ExlA (19). Surprisingly, whereas the gene *arnA* was present in all sequenced strains from other groups, only 38.5% of strains belonging to group 3 harbored the *arnA* gene, raising the question about the origins and evolution of this group of strains, as well as their mechanism of adaptation to polymyxins (16).

In this work, we showed that despite the absence of the *arn* operon, a recent clinical isolate of group 3, IHMA879472 (IHMA87) (20, 21), is capable of adaptation to polymyxins. In all investigated strains from group 3, the polymyxin-responsive gene, *IHMA87_03332* /PA1797 (renamed hereafter *mipB*), is encoded in a ParR/ParS-regulated two-gene operon together with *mipA*. We evidenced that MipA and MipB fractionate with membranes, and MipA co-purifies with MipB. Comparative proteomic analysis of envelope proteins following PMB challenge showed that the deletion of *mipBA* led to a significant decrease in efflux pump proteins MexXY and OprA, known to be regulated at transcriptional level by ParS/ParR. In addition, we showed that MipA interacts specifically with PMB/PME by nano differential scanning fluorimetry (nano-DSF). These data confirm structural predictions by AlphaFold showing MipA as a β-barrel outer-membrane protein with an internal negatively charged channel able to accommodate the PMB molecule. As the *mipBA* deletion abolished *mexXY-oprA* induction in response to PMB, we propose that MipA acts as a co-sensor of ParS-ParR regulatory system. MipA could entrap polymyxins and transmit the signal through its partner, MipB, to the inner-membrane HK sensor protein ParS, thus representing a new concept of detection for this class of antibiotics.

## RESULTS

### L-Ara4N is not essential for polymyxin adaptation

**Fig 1:**
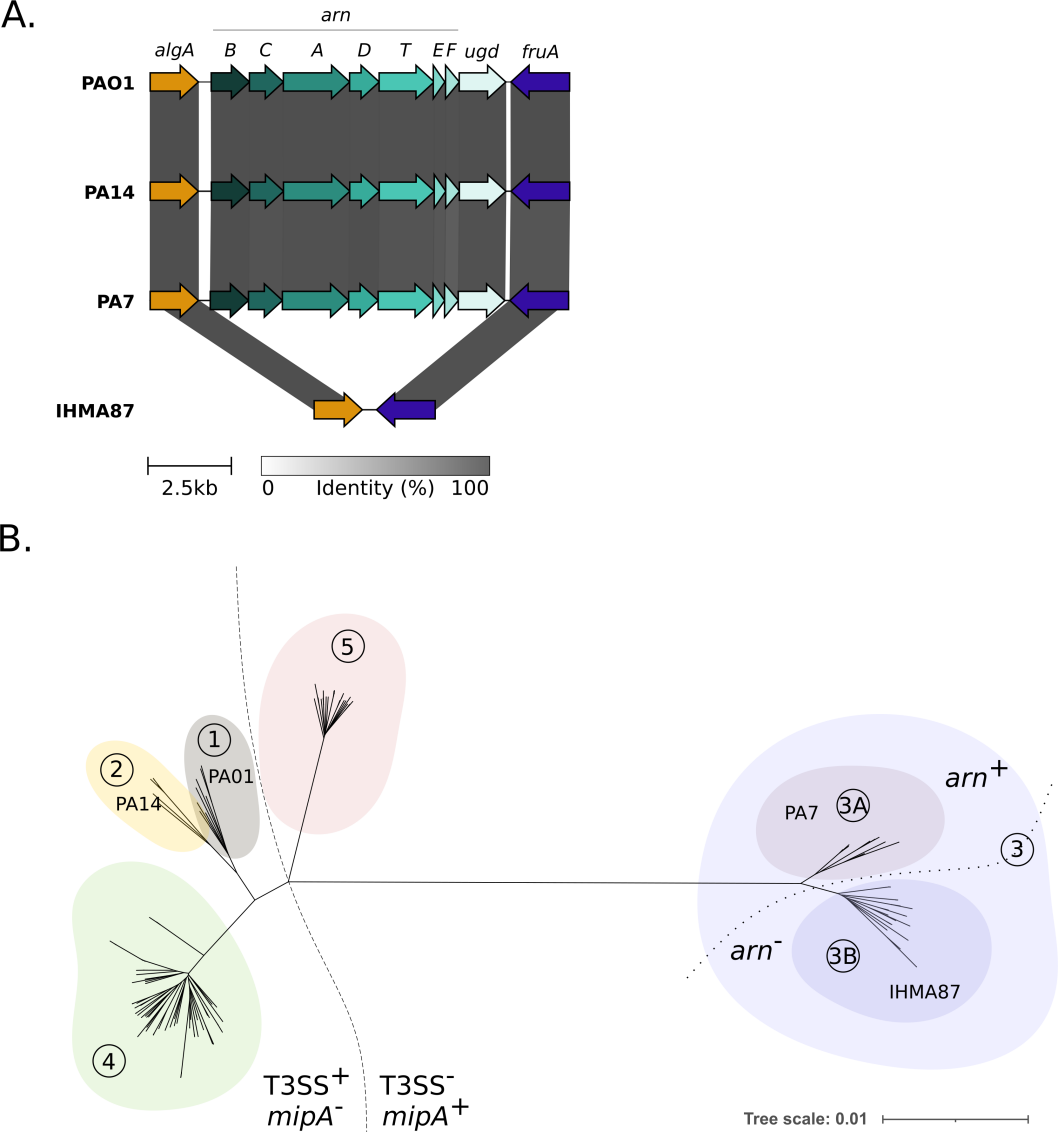
Identification of *P. aeruginosa* strains lacking *arn* operon. (A) Conservation of *arn* locus across *P. aeruginosa* strains, visualized by Clinker (23). The *arn-ugd* region expending from *algA* to *fruA* encompasses 8.8 kb and is not present in IHMA87. (B) Neighbor-joining phylogenetic tree highlighting separation of phylogenetic group 3 into two distinct subgroups, 3A and 3B, which differ notably by the presence or absence of the *arn* locus.

Unlike other species such as *Escherichia coli* and *Acinetobacter baumannii*, the addition of L-Ara4N to lipid A was sufficient to confer polymyxin resistance to both selected *in vitro* mutants and clinical strains of *P. aeruginosa* (22). The strain IHMA87 lacks the entire *arn* operon encompassing the deletion of approximately 8.8 kb between genes *algA* and *fruA*, corresponding to PA3551 and PA3560 in PAO1 (Fig. 1A). To evaluate the frequency of this event, we compared complete genomes available on the National Center for Biotechnology Information (NCBI) database belonging to groups 3 (*n* = 23), 4 (*n* = 64), and 5 (*n* = 38) with a set of strains of groups 1 (strain PA14, *n* = 8) and 2 (strain PAO1, *n* = 7). Interestingly, only a subset of strains of group 3 carried the deletion of the *arn* locus reminiscent to the IHMA87 genome (subgroup 3B), whereas 31.6% of strains (subgroup 3A, *n* = 12, strain PA7) possessed the complete *arn* operon (Fig. 1B).

**Fig 2:**
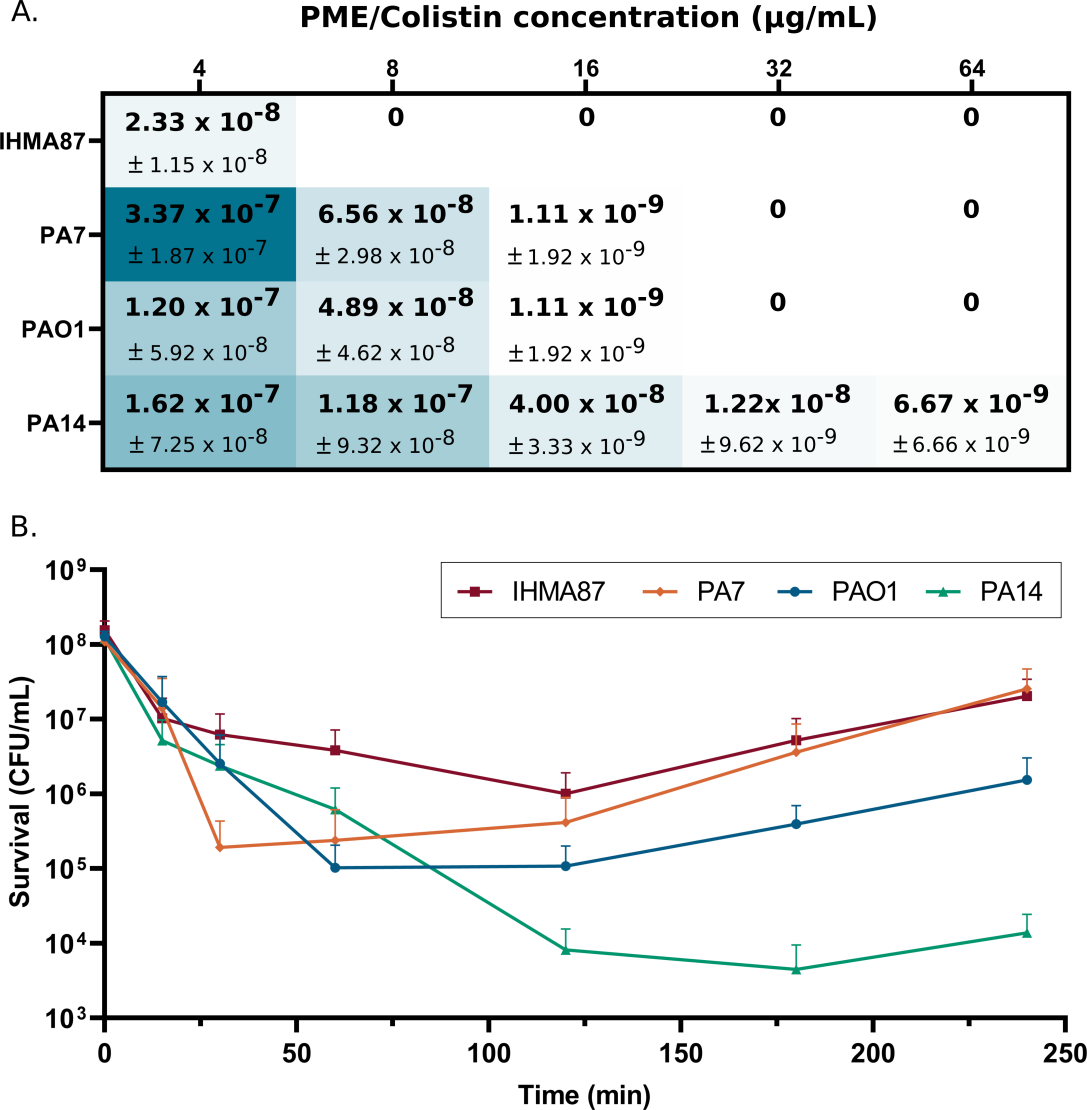
*arn* operon is necessary for acquisition of stable resistance but not for adaptive resistance to PME. (A) Frequency of acquisition of stable resistance in IHMA87 and reference strains PA7, PAO1, and PA14 in the presence of 4 to 64 µg/mL PME. (B) Bactericidal activity of PME on IHMA87, PA7, PAO1, and PA14 strains over time in the presence of 8× MIC (4 µg/mL) of PME (*n* = 3).

The minimal inhibitory concentration (MIC) of PME, toward eight selected strains of group 3B, including IHMA87, was identical to those of reference strains PAO1, PA14, and PA7 (MIC = 0.5 µg/mL) in agreement with a previous study indicating that *arn* operon is not involved in intrinsic polymyxin resistance (24). Acquired resistance to polymyxins in clinical strains of *P. aeruginosa* are associated with mutations in one or several genes encoding TCSs PmrA/PmrB, ParR/ParS, PhoP/PhoQ, and CprR/CprS, leading to constitutive *arn* operon expression and L-Ara4N addition to LPS (25, 26). We therefore evaluated the impact of this large deletion on the selection of PME-resistant mutants *in vitro*. In contrast to strains PAO1, PA7, and PA14, no resistant mutant was obtained from IHMA87 grown on Mueller-Hinton agar plates supplemented with 4–64 µg/mL of PME, showing that the *arn* operon is required to acquire stable PME resistance. We observed that according to a genetic background (PAO1, PA14, and PA7), the rates differed from 1.20 × 10^−7^ (±5.92 × 10^−8^) to 6.67 × 10^−9^ (±6.66 × 10^−9^) (Fig. 2A). Interestingly, the selection of PME-resistant mutants has proven to be unsuccessful with other clinical strains (ZW26, JT87, CPHL1145, and LMG5031) lacking the *arn* operon.

*P. aeruginosa* is also able to tolerate polymyxins in growth medium through the induction of three TCSs PmrA/PmrB, ParR/ParS, and CprR/CprS (9, 12, 27–29). To test the tolerance of the *arn* negative strain, IHMA87, the strains were exposed to step-by-step increase concentration of PME. Interestingly, as the others, the IHMA87 strain was able to grow with up to 64 µg/mL of PME, but with a lower number of viable bacteria (Fig. S1A; 2.2 × 10^4^ ± 7.7 × 10^4^ CFU/mL versus 6.7 × 10^6^ ± 1.1 × 10^6^ CFU/mL for strain PAO1, 8.3 × 10^4^ ± 3.1 × 10^4^ CFU/mL for strain PA14, and 2.4 × 10^7^ ± 3.1 × 10^7^ CFU/mL for strain PA7), whereas growth in the absence of polymyxin was comparable (Fig. S1B). Overall, these data suggest that the capacity of adaptation to PME persists in the IHMA87. To confirm that IHMA87 was able to respond to PME, we determined the bactericidal effect of supra concentrations (8× MIC) of PME. As indicated in Fig. 2B, a higher bactericidal effect was observed for the strains PAO1 and PA14 in comparison to strains IHMA87 and PA7 after 30 min post exposure. The regrowth started after 60 and 120 min were more pronounced for PA7 and IHMA87, leading to a 1–3 log difference in bacterial CFU after 240 min of treatment. The absence of L-Ara4N synthesis does not seem to alter the adaptation of the strain IHMA87 to PME. Overall, a strain devoid of *arn* operon is able to adapt to polymyxin stress, suggesting alternative *arn*-independent mechanisms in play.

### *mipBA* is activated by polymyxins in a ParR/ParS-dependent manner

**Fig 3:**
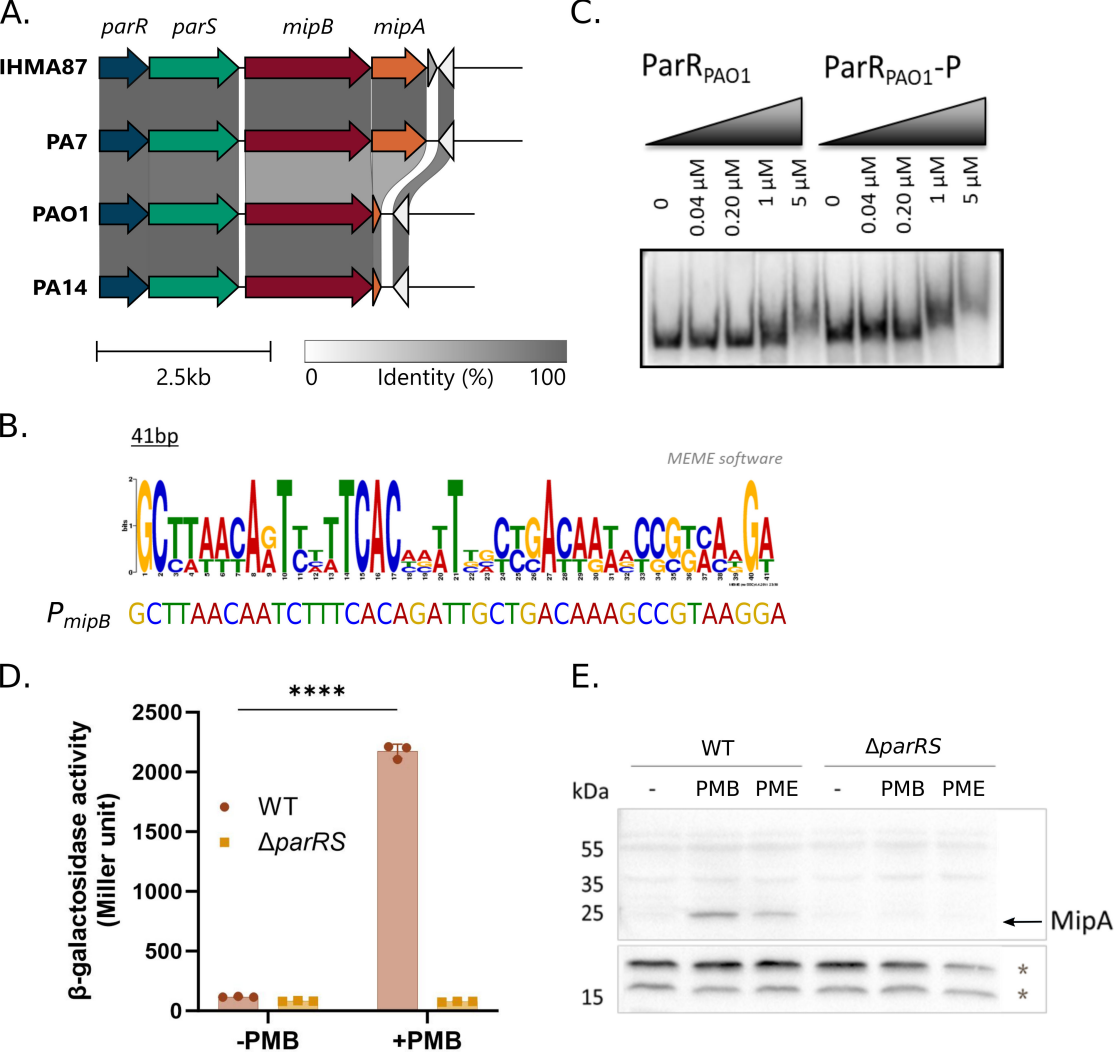
MipA is synthetized in response to PMB in a ParR/ParS-dependent manner. (A) Conservation of *mipBA* locus across *P. aeruginosa* strains, visualized by Clinker (23). (B) Consensus of ParR-binding site obtained from known target promoters and ParR-binding site in *PmipB* (PA1797). (C) Electrophoresis mobility shift assay showing ParR binding onto the promoter of *mipB*/*PA1797* with or without phosphorylation (P). (D)*P_mipBA_* activity in response to PMB measured by β-galactosidase assay in wild type (WT-IHMA87) and IHMA87Δ*parRS* mutant. ****: *P* < 0.0001. (E) MipA detection in response to sub-lethal concentrations of PMB and PME in a ParR/ParS-dependent manner. * indicates non-specific antibody binding used as loading control. PMB concentration: 0.25 µg/mL.

In order to investigate the possible role of other genes in PMB adaptation, we focused on the gene of unknown function, *IHMA87_03332* /PA1797 (renamed *mipB*), which is induced by PMB in a ParR/ParS-dependent manner (9, 12). The genetic environment of *mipB* across *P. aeruginosa* strains is variable (Fig. 3A). Although the synteny of the *parRS* operon and *mipB* is conserved across the clades, strains of group 3 harbor a second gene immediately downstream of *mipB* annotated as *mipA* for MltA-interacting protein A (Fig. 3A). In PA14 and PAO1, the predicted open reading frame of residual *mipA* is 117 nucleotides long compared to the 780-bp *mipA* gene in IHMA87 and PA7. The residual predicted protein MipA* shares more than 60% identity over 38-amino acid-long sequences, suggesting a genetic remodeling of the region and partial loss of *mipA* sequences. tRNA coding sequences, frequently found as hotspots for genetic remodeling (30, 31), are present just downstream of *mipA* (*Pseudomonas* genome database [32]).

As the ParR regulon has been largely investigated (9, 12, 33), the ParR-binding consensus sequence could be defined using the bioinformatics tool MEME. A perfect ParR-binding site, conserved across the reference strains, was identified upstream the ATG of *mipB* (Fig. 3B). We examined whether the regulation of *mipB* is direct, using purified ParR protein and electrophoresis mobility shift assay (EMSA) on a predicted promoter region of *PA1797*/*mipB*. In agreement with the recent genome-wide binding pattern of ParR (33), we showed a direct binding of ParR to the promoter of *PA1797*, which was improved when ParR was phosphorylated (Fig. 3C). We then used reverse transcription quantitative PCR (RT-qPCR) to re-examine the expression and predicted operonic structure of *mipBA* in IHMA87, in the presence of PMB. The polycistronic RNA *mipB-mipA* was produced in high amounts in response to PMB (30-fold increase), confirming that the two genes form a PMB-responsive operon (Fig. S2A). To confirm the role of ParR in *mipBA* gene expression, we designed a transcriptional reporter and measured *P_mipBA_* promoter activity in response to a sub-lethal dose of PMB (0.25 µg/mL). As shown in Fig. 3D, *P_mipBA_* activity was increased 18-fold in response to a sub-lethal PMB treatment in a ParR/ParS-dependent manner. A dose-response effect of PMB on *P_mipBA_* activity was observed increasing up to 0.3 µg/mL PMB before reaching saturation (Fig. S2B).

We then examined the levels of MipA synthesis in response to PMB using specific polyclonal antibodies. MipA was detected in response to sub-lethal PMB treatment and was undetectable in a *parRS* deletion mutant (Fig. 3E). Using MipA-directed antibodies and a version of MipB with 3× FLAG tag at the C-terminus (MipB_3× FLAG_), we followed the kinetic of MipB_3× FLAG_ and MipA production upon the addition of a sub-lethal concentration of PMB. MipB_3× FLAG_ was detected from 15 min after the addition of PMB (Fig. S2C), and the quantities of MipB_3× FLAG_ increased up to 60–90 min, respectively. MipB was not detected in late stationary phase. Low amounts of MipA were detected 30 min after PMB addition, reached a maximum at 90 min, and was still detectable in late stationary phase. These results show that both MipA and MipB_3× FLAG_ are rapidly produced in response to PMB treatment and reached their maximum abundance between 60 and 120 min, with MipB_3× FLAG_ being less stable. To determine whether PMB-dependent induction of MipA was conserved in other T3SS-negative strains, we used nine isolates from groups 3 and 5 (34) (Fig. S2D). Overall, MipA induction in response to sub-lethal concentration of PMB appears to be a conserved mechanism except for the strain DVL1758, in which the protein could not be detected. Interestingly, in one strain, Zw26, MipA was constitutively produced, which probably results from a constitutive activation of the ParS/ParR system due to mutations (35).

Overall, the ParR-dependent production of MipA and MipB is triggered by a sub-lethal concentration of PMB and PME and is conserved across representative strains from groups 3 and 5 of *P. aeruginosa*.

### MipA and MipB are associated with the bacterial membranes

**Fig 4:**
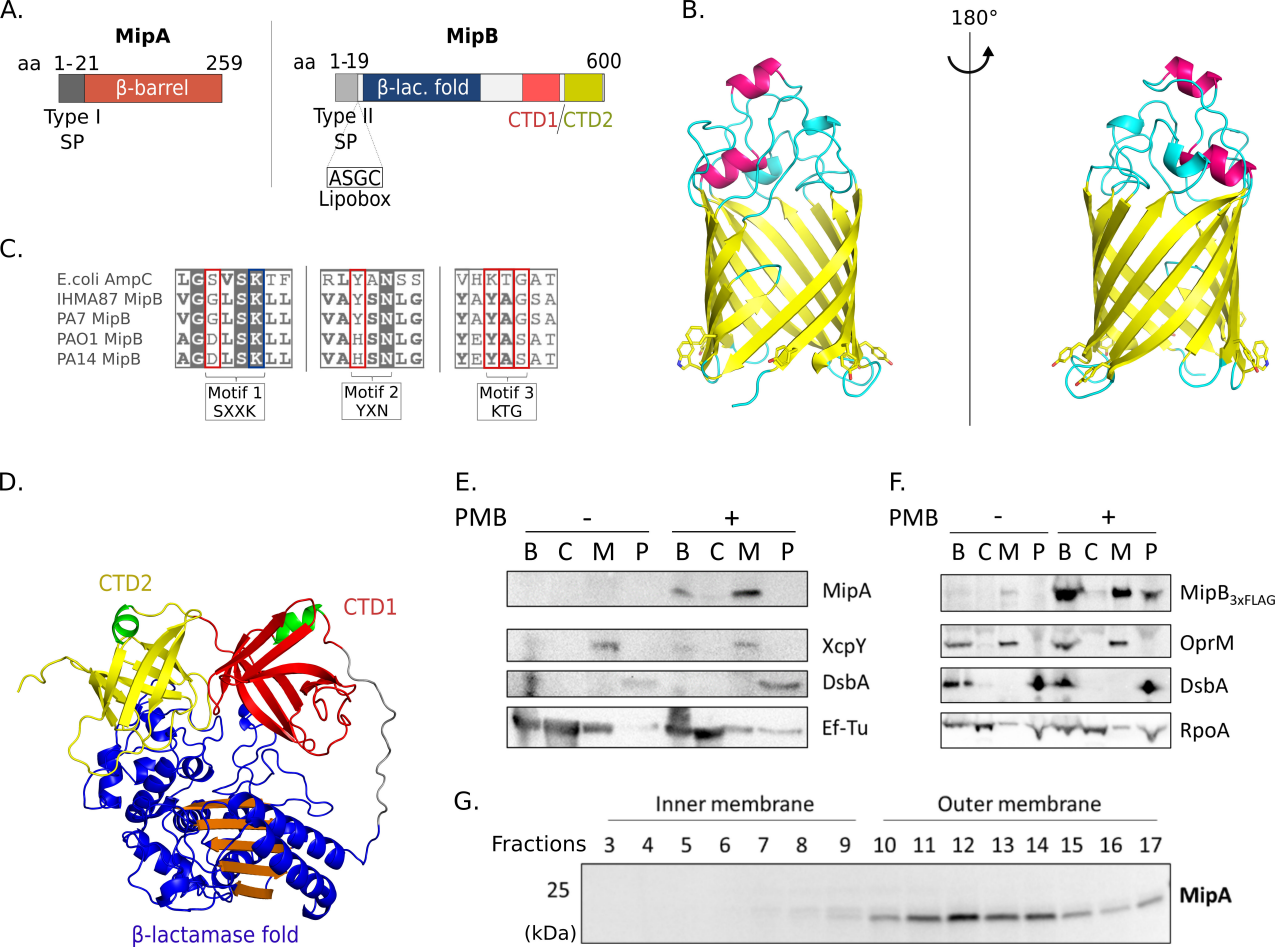
MipA and MipB are envelope proteins. (A) Schematic representation of MipA and MipB. Both proteins carry N-terminal signal peptides predicted by SignalP v.5.0 (45). MipB contains an additional “lipobox” sequence composed of “ASGC” sequence with conserved cysteine residue, which anchors proteins to the membrane. (B) MipA model generated by AlphaFold (46), without the 21 first residues containing the signal peptide. The β-strands are represented as yellow arrows, and α-helices are represented as pink ribbons. The side chains of aromatic residues that delineate the inner membrane are drawn in sticks. Figures were generated with Pymol. (C) Alignment of the catalytic sites of AmpC (*E. coli*) with MipB from different *P. aeruginosa* strains (IHMA87, PA7, PAO1, and PA14). (D) MipB model calculated using AlphaFold (46) excluding the 27 first and 8 last residues. The β-strands are represented as arrows, and α-helices are represented as ribbons. The N-terminal domain containing the β-lactamase fold is in blue and orange. The two eight-stranded anti-parallel β-barrels in the C-terminal are in red and yellow, and their short N-terminal α-helices are in green. The long loop connecting the C-terminal to the N-terminal domain is in gray. (E) Bacterial fractionation showing membrane association of MipA. XcpY, DsbA, and Ef-Tu includes controls for membranes, periplasm, and cytosolic fractions, respectively. (F) MipB_3xFLAG_ fractionates with the membranes and, to a lesser extent, with the perisplam. OprM, DsbA, and RpoA are controls for membranes, periplasm, and cytosolic fractions, respectively. (G) Inner and outer-membrane separation by sucrose gradient showing the presence of MipA in the outer-membrane fractions. PMB concentration: 0.25 µg/mL. B, bacteria; C, cytosol; M, membrane; P, periplasm.

MipA and MipB are predicted to localize to the bacterial envelope. MipA, which belongs to the MipA/OmpV family, harbors an N-terminal signal peptide (M1-A21) predicted to be cleaved according to SignalP v.5.0 (Fig. 4A) (36). Using AlphaFold (37), we generated a structural model of MipA (Fig. 4B), which folds as a β-barrel with 12 transmembrane β strands. The presence of five tryptophan residues (four of them highly conserved in MipA proteins, Fig. S3) suggests that the β-barrel membrane insertion occurs via the same mechanism as observed for OmpA, without the need of Bam complex (Fig. 4B) (38). The 12 β-strands are connected by long extracellular loops that close the lumen of the barrel and periplasmic turns, given the localization of the N- and C- termini on the same side (38). The lumen of the barrel is opened on the periplasmic space and on the side through a lateral gate delineating a channel that connects the periplasm to extracellular space. The β-barrel has a cross-section of ∼25 Å × 22 Å and is ∼40 Å in height. Using PDBeFold (39) and FoldSeek (40), MipA structure superimposes on several structures from PDB such as OmpG (PDB entry: 2 × 9K [41]), and NanC (PDB entries: 2WJR and 2WJQ [42]) with root-mean-square deviation (rmsd) values of 2.55 and 2.58 Å, two porins from *E. coli* involved in the N-acetylneuraminic and maltodextrin transport, respectively. MipA also superimposes onto the barrel domain of the intimin protein from *E. coli* (PDB entry: 5G26 [43]) and the lipid A deacylase LpxR of *Salmonella* Typhimurium (PDB entry: 3FID [44]) with rmsd values of 2.33 and 3.01 Å, respectively. Using FoldSeek, several predicted structures by AlphaFold as β-barrels with 12 transmembrane β-strands and the lateral gate superimpose onto MipA; these proteins belong to different species of Gram-negative bacteria.

On the other hand, MipB carries a type II signal peptide (residues M1–G21) and an “ASGC” lipobox sequence characteristic of bacterial lipoproteins (Fig. 4A; Fig. S4). The amino acids at positions +2 and +3 after the Cys suggest that MipB is targeted to the outer membrane (47, 48). MipB harbors a β-lactamase fold but lacks the “SXXK” motif necessary for activity (Fig. 4C) (49). MipB also shows degenerated motif 2 (YXN) and motif 3 (KTG), present in the *E. coli* K12 β-lactamase AmpC (Fig. 4C) (50, 51). In addition, MipB possesses 220 additional residues at its C-terminus that are absent in *E. coli* AmpC, suggesting this domain may provide specific function to *P. aeruginosa* MipB.

Using AlphaFold (37), we also generated a model of MipB (Fig. 4D). As suggested by sequence alignment (Fig. S4), MipB harbors a “serine” β-lactamase-like fold (residues M28–S387) with a central five-stranded anti-parallel β-sheet surrounded by α-helices similar to the structure of β-lactamases and penicillin-binding proteins (PBPs) (52, 53). Two additional C-terminal domains, CTD1 (V405–I501) and CTD2 (L506–R593), folded as an eight-stranded anti-parallel β-barrel, are connected to the main domain by a linker formed by a long loop (G388–A404). Both are closed by a short N-terminal α-helix (Fig. 4D). Interestingly, CTD1 and CTD2 are conserved in MipB proteins in strains of three clades independently of the presence of MipA (Fig. S4). These two domains have folds similar to the C-terminal domain of penicillin-binding protein Rv0907 from *Mycobacterium tuberculosis* H37RV (PDB 4WHI [54]) of unknown function. The analysis of the electrostatic potential of these two domains shows a large, positively charged surface exposed to the external part of the protein.

In order to experimentally corroborate the localization of the two proteins, we used a bacterial fractionation method to separate the bacterial cytoplasm, periplasm, and membranes and investigate protein partitioning in bacteria following the PMB challenge. The immunoblotting performed on the different fractions confirmed the fractionation of MipA and MipB with bacterial membranes (Fig. 4E and F), with one fraction of MipB also detected in the periplasm, in agreement with export sequence signals. We further separated the inner and outer membranes on a sucrose gradient (55, 56) and confirmed that MipA is associated with the outer membrane (Fig. 4G), in line with the structural predictions. As MipB has no predicted transmembrane domains, its membrane association may be mediated by the lipid anchor and through association with MipA.

**Fig 5:**
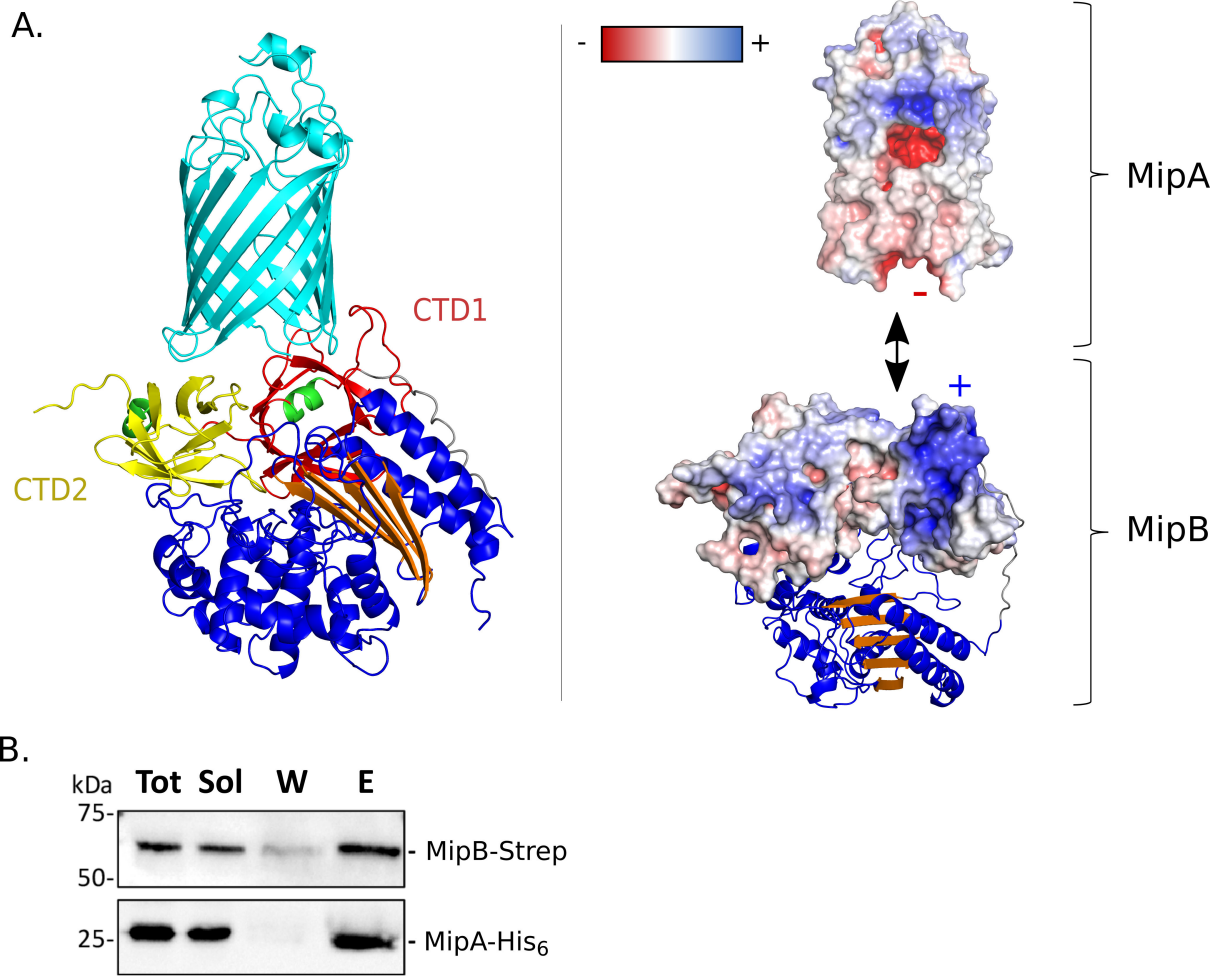
MipA and MipB interaction. (A) Model of the MipA-MipB complex generated using AlphaFold-Multimer (46). (Left) MipA is in cyan. The β-lactamase fold of MipB is in blue and orange. The two eight anti-parallel β-stranded barrel domains of MipB are in yellow and red with the first α-helices in green. (Right) Electrostatic surface representation showing the charges at the interface between the two proteins. The figures were generated with Pymol. (B) MipA and MipB co-purify *in vitro*. MipB-Strep and MipA-His_6_ were co-produced in *E. coli*. Soluble extracts were loaded onto a strep column, and proteins were eluted by the addition of a desthobiotin-containing buffer. Different fractions (Tot, Sol, W, and E) were analyzed by immunoblotting using anti-Strep-tag and anti-MipA primary antibodies. E, elution; Sol, soluble extract; Tot, total extract; W, washing.

To investigate potential interaction between the two proteins, a complex between MipA and MipB was built using AlphaFold-Multimer (46). Its analysis reveals two main interaction zones that encompass the eight-stranded anti-parallel β-barrels of the MipB CTDs that interact with the open-periplasmic side of MipA (Fig. 5A), mainly through charged residues. To confirm the relevance of this structural model, we co-produced MipB and MipA in *E. coli* and performed an affinity chromatography experiment. Proteins were produced in *E. coli* from a bicistronic vector yielding MipA-His_6_ and MipB-Strep. Due to their membrane localization, we tested the effective solubility of both proteins in the presence of different detergents. The highest solubility was obtained in the presence of N-lauroylsarcosine. MipB-Strep and its potential partner were purified by affinity chromatography on a Strep column. The obtained fractions were analyzed by immunoblotting (Fig. 5B). The eluted fraction contained both MipA-His_6_ and MipB-Strep showing that the two proteins co-purify. Of note, MipA-His_6_ alone did not bind to the Strep column (Fig. S5), implying that MipA interacts with MipB.

### MipA specifically binds PME and PMB

**Fig 6:**
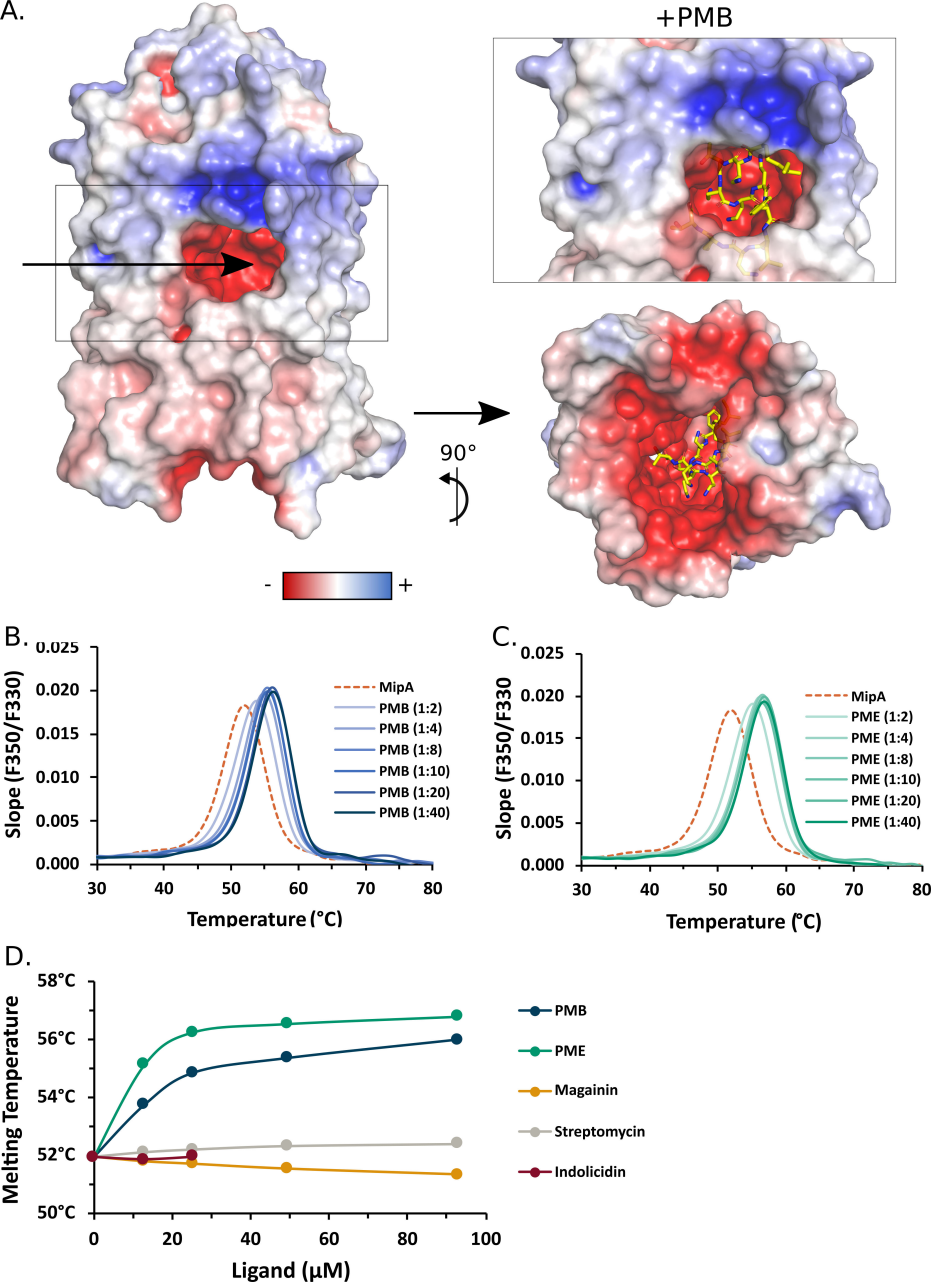
MipA specifically binds PMB and PME. (A) PMB docking in MipA. (Left) MipA electrostatic surface. The arrow shows the negative channel entrance. (Right top) Membrane perpendicular view from the PMB bound to MipA. The best position of PMB, calculated with Autodock Vina (46), is shown in sticks: the carbon, nitrogen, and oxygen atoms are in yellow, blue, and red, respectively. (Right bottom) View from the MipA periplasmic face of the PMB bound to MipA. (B and C) MipA thermal stability increases in the presence of PMB and PME as assessed by nano-DSF. Pure MipA alone (in orange, dashed line) or incubated for 2 h at room temperature with a molar ratio from 1:2 to 1:40 of PMB (B) or PME (C) was heated from 20°C to 95°C. Protein folding/unfolding was followed by tryptophan fluorescence emitted at 330 and 350 nm. The slope of the ratio (F350:F330) was plotted at different temperatures, and its maximum corresponds to the melting temperature (Tm) of the protein. (D) Summary of calculated Tm of MipA in presence of PMB, PME and three other positively charged molecules, magainin, indolicidin, and streptomycin, used as negative controls, assessed by nano-DSF. Data from panels B and C was also included for comparison. Indolicidin was used only up to 1:4 molar ratio, which corresponds to 25 µM. Of note, indolicidin contains five tryptophans and could not be used at higher concentrations because of the high background signal.

The electrostatic surface analysis of MipA shows that the protein is hydrophobic on the outside of the β-barrel, the hydrophobic membrane part being delineated by a girdle of aromatic residues at the periplasmic interface, coherent with a membrane-embedded protein. Interestingly, the channel of MipA is mainly composed of negatively charged amino acids (Fig. 6A, left) that together with its size are compatible with the binding of positively charged molecules such as polymyxins. We therefore tested this hypothesis by docking PMB and PME into MipA using Autodock Vina (57) and found highly confident solutions (Fig. 6A, right), suggesting that MipA may bind PMB and PME.

The possible interactions between MipA and PMB/PME were examined by DSF, a biophysical method of choice to investigate low-molecular-weight ligands binding to proteins employing intrinsic tryptophan fluorescence (58). Here, we performed nano-DSF to determine protein stability in the presence of cAMPs. MipA, purified to homogeneity (Fig. S6A), was mixed with PMB or PME at different molar ratios from 1:1 to 1:40 (MipA:ligand), corresponding to up to 200 µM of the ligands (Fig. 6B and C). Samples were incubated at room temperature for 2 h and then analyzed by nano-DSF to determine the apparent melting temperature (Tm) and the dissociation constant (*K*_D_), as a measure of protein folding/unfolding and stability. In the purification buffer containing 0.1% LAPAO, MipA displayed a Tm of 51.9°C. Upon addition of PMB or PME, the Tm values increased to 56.2°C and 56.8°C, respectively (Fig. 6B and C), inferring binding to MipA. Three positively charged ligands used as negative controls—magainin II, indolicidin, and streptomycin (Fig. 6D)—as well as the buffer alone (Fig. S6B) did not modify the initial Tm of MipA, indicating specificity of the PMB/PME-MipA interaction (Fig. 6B and C). A similar shift in Tm of 6°C was observed previously upon PMB binding to LSD1 demethylase (59). The binding data were consolidated by the calculation of a *K*_D_ for both ligands (*K*_D_ of 8.4–10.0 µM for PMB and 2.8–3.4 µM for PME) using FoldAffinity online tool (60, 61) (Fig. S7). Together, these results show a stabilization of MipA in the presence of both cAMPs and demonstrate a direct and specific binding of the two molecules to MipA, in agreement with docking data.

### *mipBA* deletion alters bacterial response to polymyxins

**Fig 7:**
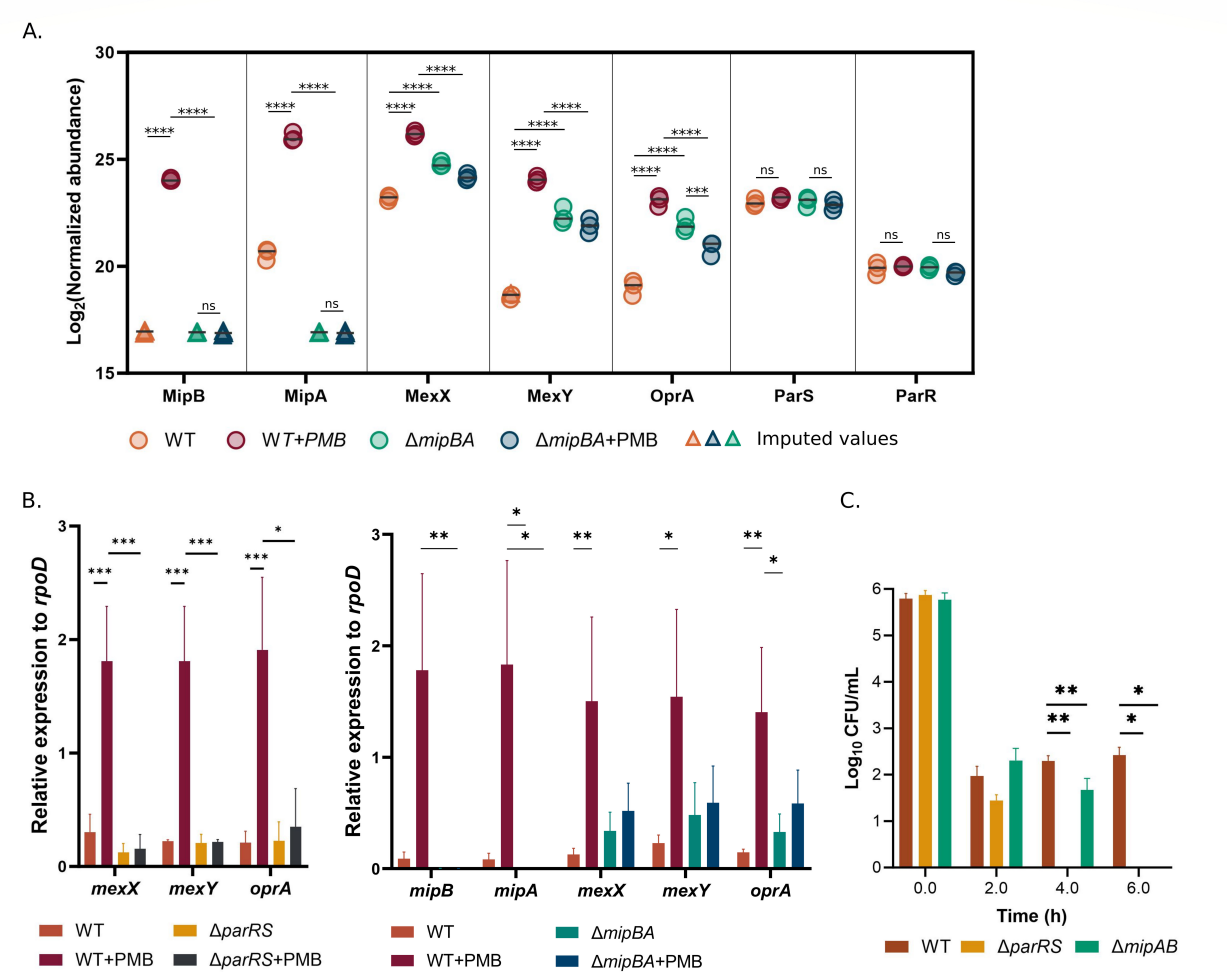
MipA/MipB are required for *P. aeruginosa* response to polymyxins. (A) Normalized abundance of MipA, MipB, MexX, MexY, and OprA in IHMA87 (WT) or Δ*mipBA* membranes with or without sub-lethal PMB treatment (0.25 µg/mL) obtained by proteomic analysis (*n = 3*). Data represented by a triangle were imputed using the slsa algorithm for partially observed values in the condition and the DetQuantile algorithm for totally absent values in the condition. (B) Relative expression level of *mipB*, *mipA*, *mexX*, *mexY*, and *oprA* in WT and Δ*parRS* (left) or IHMA87 (WT) and Δ*mipBA* (right) with or without addition of sub-lethal concentration of PMB (0.25 µg/mL) normalized to *rpoD* by RT-qPCR. A Kruskal-Wallis test was applied followed by a Dunn test for each of the genes tested. Note the absence of *mex* induction in Δ*parRS* and Δ*mipBA*. (C) Bactericidal effect of PME/colistin used at 1 µg/mL on cultured of IHMA87 (WT), Δ*mipBA*, and Δ*parRS* over time (*n = 3*). *: *P* < 0.05, **: *P* < 0.01, ***: *P* < 0.001, ****: *P* < 0.0001.

Taking into account the envelope localization of MipA and MipB and polymyxin binding to MipA, we hypothesized that MipA/MipB work in concert to defend the bacterial cell against polymyxin-induced envelope damage. We adopted a mass spectrometry-based quantitative proteomic analysis of bacterial membranes to investigate bacterial response to PMB through the comparison of protein abundances in a Δ*mipBA* mutant compared to the parental strain (Table S1). In the wild-type IHMA87 strain, treatment with a sub-lethal concentration of PMB led to the drastic increase of both MipA and MipB in bacterial membranes (Fig. 7A), in agreement with induction of the operon by PMB on the transcriptional level and increased quantities of both proteins by immunoblotting. MipA was detected in the membranes of the wild-type strain even in basal condition without PMB treatment (undetectable by immunoblot; Fig. 3E). In addition to Mip proteins, the quantities of the proteins building the tripartite efflux pump MexXY-OprA (IHMA87_03098-IHMA87_03100) were increased (with log_2_ fold change [FC] of 3.0–5.4; Table S1), in line with *mexXY-oprA* overexpression upon PMB treatment (9, 12). Interestingly, the comparison of proteomes upon PMB treatment showed a significant decrease of the three proteins of the MexXY-OprA system in the membranes of Δ*mipBA* [log_2_(FC) ranging between −2.0 and −2.2; Fig. 7A].

The *mexXY* and *oprA* transcriptional activation in the presence of PMB depends on ParR/ParS signaling (9, 12). The fact that MipA/MipB affected MexXY-OprA quantities suggests that they could assist ParR/ParS in the sensing of polymyxins to activate the downstream signaling pathway leading to the induction of ParR regulon responsible for the adaptive resistance to polymyxins. It is worth noting that the quantities of ParR and ParS were identical in the two conditions, and similar in the two strains (wild type versus ∆*mipBA*), in agreement with previous studies showing that PMB does not transcriptionally activate the *parRS* operon (Fig. 7A) (9).

As the induction of *mexXY-oprA* upon PMB treatment occurs at the transcription level, we validated our data using RT-qPCR on bacterial cultures challenged with PMB (Fig. 7B). Indeed, the three genes of the operon *mexXY-oprA* in IHMA87 were induced by PMB, in a ParR/ParS-dependent manner, as already documented for other strains (Fig. 7B, left) (9, 12). In the *mipBA* mutant, this upregulation was abolished, suggesting that MipB/MipA participate in transcriptional regulation (Fig. 7B, right). Interestingly, although not significant, higher levels of *mex* transcripts could be measured in the *mipBA* mutants in basal conditions (without PMB), in agreement with the comparative proteomics data. It is tempting to speculate that in the absence of the signal, the low levels of MipA maintain the ParR/ParS system in an inactive form, keeping the ParR regulon shutdown.

To assess the impact of MipA/MipB on the bactericidal effect of PME, we determined the number of survivors in the IHMA87 strain, ∆*parRS* and ∆*mipBA* mutants exposed to 1 µg/mL of PME for 6 h. Consistent with our previous data (35), the number of survivors was significantly reduced for the ∆*parRS* mutant starting from 4 h. Furthermore, the effect of PME was significantly more pronounced in the ∆*mipBA* mutant compared to the parental strain after 4 h of exposure (Fig. 7C).

Overall, the absence of MipB/MipA led to a dysregulation of the PMB adaptive response, in agreement with their induction by PMB, outer-membrane localization, and structural predictions.

## DISCUSSION

While the *arn* operon has been widely studied and is well known for its contribution to polymyxin resistance in *P. aeruginosa*, clinical isolates lacking the *arn* operon have been poorly reported. In this study, we have shown that a sub-group of isolates from respiratory and urine samples lacks the entire *arn* operon. Interestingly, although *arn* operon is required to acquire stable resistance to polymyxins, adaptive resistance to polymyxins persisted in those strains, suggesting that other genetic determinants were involved.

Exposure to sub-inhibitory concentrations of PMB in the *P. aeruginosa* IHMA87 strain strongly induced the expression of *mipBA* operon through a TCS, the inner-membrane HK, ParS, and the RR, ParR. At least three TCSs (PmrAB, ParRS, and CprRS) participatd in the response to polymyxins in *P. aeruginosa* with partially overlapping regulons (9, 12). However, ParR was the only response regulator binding to *mipBA* promoter in all three strains tested (PAO1, PA14, and IHMA87) (33).

MipA and MipB proteins are associated with the membrane and co-purify when expressed in *E. coli*. MipA localization in *Caulobacter crescentu*s was dependent on MreC, a proposed scaffold protein in bacterial elongasome (62, 63). Moreover, MipA was proposed to be tethered to both outer and inner membranes through interaction with MltA and PBP1b (64). In *P. aeruginosa*, MipA appears to be embedded in the outer membrane, in line with its fold in β-barrel containing 12 β-strands that superimposes on the NanC and OmpG porins (41, 42). Structural predictions showed that MipA harbors a negatively charged inner channel in its center, where several conformations of PMB and PME molecules can be docked, reminiscent of the crystal structure of the LSD1-CoREST bound to PMB (PDB entries: 5L3F and 5L3G [59]). Further structural studies are essential to reveal the binding mode of these ligands to MipA. The MipA fold is observed in other proteins of Gram-negative bacteria, suggesting that other outer-membrane porins could bind positively charged molecules such as PMB or PME if the lateral gate and barrel lumen forming a negatively charged channel are conserved.

**Fig 8:**
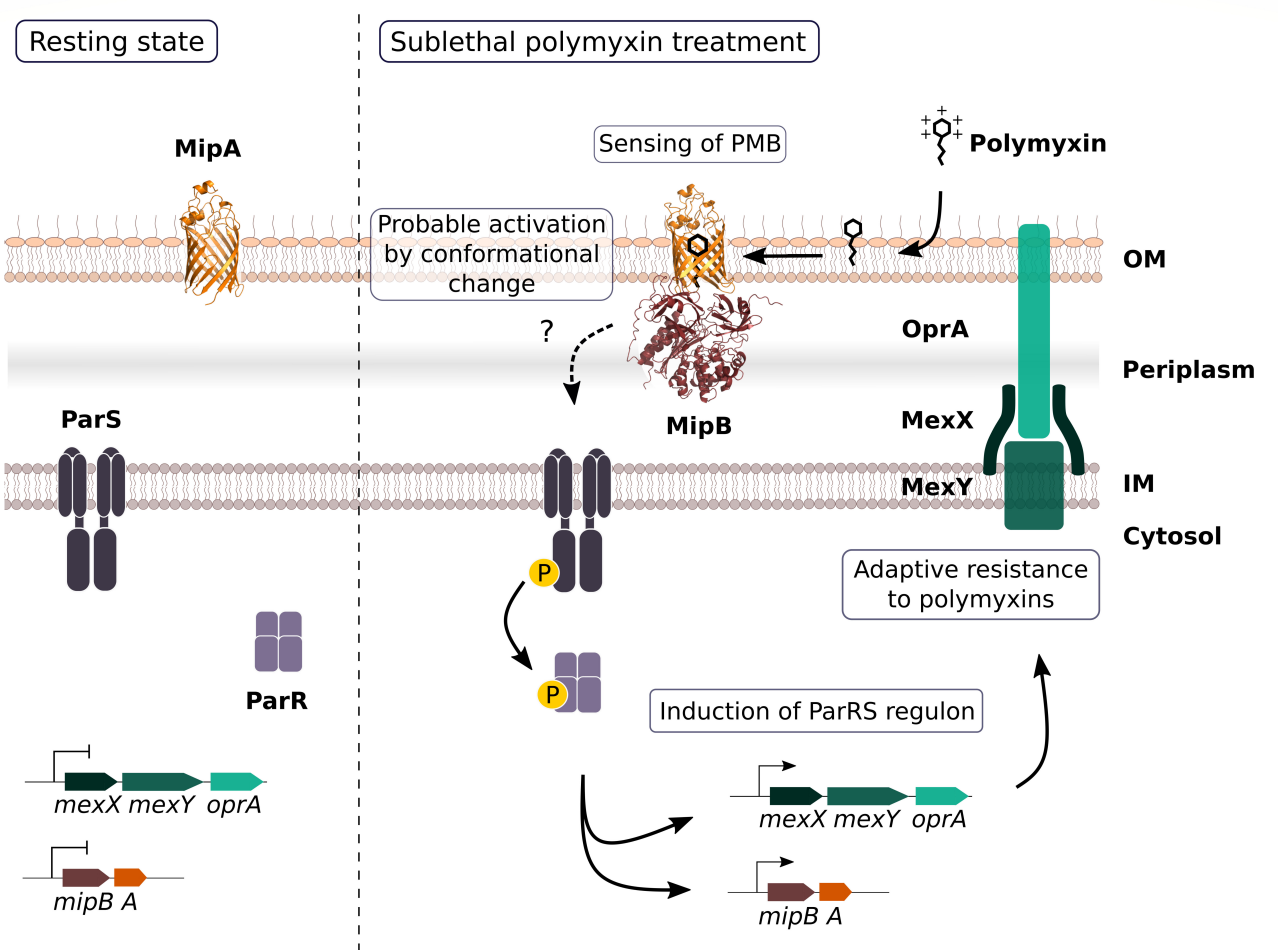
Schematic representation of the working model proposing MipA/MipB as co-sensors of PMB. In the resting state, low amounts of MipA are present in the outer membrane, and ParR/ParS TCS is inactive. PMB binding to bacterial membranes and to MipA provokes conformational changes in the MipB-MipA complex. MipA and MipB induce ParS autophosphorylation, which leads to activation of the cognate response regulator ParR. ParR binds to promoter regions of *mexXY-oprA* and *mipBA* operons, resulting in MipA, MipB, and MeXY-OprA overproduction and adaptive resistance to polymyxins.

In light of structural characteristics of Mip proteins and the finding that the *mipBA* deletion phenocopies the *parRS* mutant phenotype in the presence of PMB and PME (i.e., absence of transcriptional activation of *mexXY-oprA* and altered response to PME), we propose the model where the binding of polymyxins to MipA (potentially through conformational change in MipA-MipB interaction) initiates the downstream signaling through the kinase activity of ParS, leading to the activation of ParR/S TCS. This would allow the transcriptional activation and synthesis of the MeXY-OprA pump and the adaptive resistance to PMB (model presented in Fig. 8). ParS is a classical HK composed of two transmembrane α-helices, a 102-amino acid-sensing periplasmic domain and a cytoplasmic kinase domain. ParS belongs to the family of HKs sensing cAMPs; however, how the sensing occurs at the molecular level is unknown. There is scarce structural information concerning the recognition of and signaling initiation by HK, probably due to their size, membrane localization, and dynamics of the phosphate transfer (for review, see reference 65). A recent high-resolution structure of a nitrate/nitrite-sensing histidine kinase, NarQ, revealed how the mechanistic signal can be amplified and propagated through the protein leading to kinase activity (66). For example, it is established that the HK PhoQ of *Salmonella* is activated by cAMPs through displacement of magnesium ions (Mg^2+^) (67). Therefore, small conformational changes in the perception domains of the sensing protein may lead to adapted transcriptional response.

Co-sensing or signal transfer between macromolecules occurs in several signal transduction TCSs in Gram-positive bacteria (for review, see reference 68). The most studied system is the *Bacillus subtilis* Bce module providing anti-microbial peptide resistance. This stress envelope response module is composed of an ABC transporter, BceA/BceB, which forms a complex with the inner-membrane sensor kinase, BceS, of the TCS BceS/BceR (69, 70). Architecture of a complete Bce module by cryo-EM revealed extensive structural flexibility of the kinase BceS upon the bacitracin-dependent ATP binding to the ABC transporter, BceAB (71).

Moreover, the MipA/MipB-ParR/ParS module shares striking functional and structural analogy with *E. coli* surface sensing module composed of an outer-membrane lipoprotein NlpE that interacts with OmpA (72) acting as a membrane sensor of the envelope stress response TCS CpxR/CpxA (73). The flexible N-terminal domain of NlpE interacts directly with the periplasmic domain of the CpxA kinase (74, 75) to initiate the downstream phosphorelay. Indeed, using PDBeFold (39), we superimposed CTD2 of MipB onto the β-barrel M21-M99 of the sensor liporotein NlpE with an rmsd value of 2.22 Å. Investigating detailed molecular interactions between MipA/MipB and ParS will be a challenge of our future work.

Using the *arn* operon as a readout, Fernández et al. showed that the ParR/ParS TCS in *P. aeruginosa* responds to a variety of cAMPs, with the best inducer being indolicidin, followed by PMB (9). They also showed that the inactivation of *mipB* by transposon insertion in PAO1 leads to reduced adaptive resistance to PMB (9); however, how the signaling occurs without the complete MipA is difficult to apprehend, although we cannot exclude that structural homologs of MipA exist in the PAO1 group of strains. Despite the fact that indolicidin was able to activate ParR/ParS signaling (9), it did not bind to and stabilize MipA in nano-DSF experiments (Fig. S6), suggesting alternative cAMPs sensing mechanisms able to activate ParR/ParS.

The study of *mipA* genetic environment across Gram-negative bacteria performed by WebFlaG (76) highlighted the presence of MipA-like outer-membrane proteins in many distinct species; however, its genetic association with *mipB* is present only in species from the *Pseudomonas* genus (genetic neighbor 3; Fig. S8). It is worth noting that a gene coding for another shorter serine hydrolase domain-containing protein was found next to *mipA* in the *Achromobacter* genus. Interestingly, the screen of *mipA* loci revealed that, in most cases and even in the absence of *mipB*, a TCS was encoded just upstream or downstream of *mipA*, further suggesting a role of MipA in signaling and/or stress response (genetic neighbors 1 and 2; Fig. S8).

Interestingly, in the strain devoid of the *arn* locus, the only membrane proteins induced by PMB were MexX, MexY, and OprA, forming the resistance-nodulation cell division-type efflux pump MexXY-OprA (77). The outer-membrane protein OprA is lacking in PAO1 genetic background, and the periplasmic MexX protein and cytoplasmic membrane protein MexY function in cooperation with the outer-membrane protein OprM (78). This active efflux pump has a wide substrate specificity (but not polymyxins) and contributes to intrinsic and acquired resistance to aminoglycosides in *P. aeruginosa* strains (79). MexXY overproduction is very frequent in clinical strains from health-associated infections and cystic fibrosis (80, 81). *mexXY* belongs to the ParR regulon and, together with ParR/ParS-upregulated genes of the polyamine biosynthetic pathway (PA4773-PA4774-PA4775), contributes to PME/colistin tolerance and adaptive resistance (35). The molecular mechanism leading to PMB protection by MexXY-OprA is still unknown, and the IHMA87 strain represents the opportunity to investigate this aspect in the *arn*-negative genetic background.

Altogether, we provide new insights into *P. aeruginosa* global response to polymyxins and the heterogeneity among *P. aeruginosa* strains. We show that a strain devoid of *arn* operon is capable of adaptation to polymyxins, and we propose that MipB-MipA constitutes a new sensor of polymyxins, which may signal outer-membrane perturbation to HK ParS, of the ParR/ParS two-component signaling system, to orchestrate the bacterial response to PMB and probably other anti-microbials targeting outer membranes.

## MATERIALS AND METHODS

### Bacterial strains and genetic manipulations

*E. coli* and *P. aeruginosa* were grown in lysogeny broth (LB) at 37°C under agitation. *P. aeruginosa* was selected on irgasan (25 µg/mL)-containing LB plates. Antibiotics were used at the following concentrations: 75 µg/mL gentamicin, 75 µg/mL tetracycline, and 100 µg/mL carbenicillin for *P. aeruginosa* and 50 µg/mL gentamicin, 10 µg/mL chloramphenicol, and 100 µg/mL ampicillin for *E. coli*. Unless specified otherwise, *s*ub-lethal concentration of PMB used for *P. aeruginosa* strains was 0.25 µg/mL. All strains and plasmids are listed in Table S2.

Plasmids for protein expressions pET15b-VP-*mipB-Strep/mipA-His*_6_ and pET15b-VP-*mipA-His*_6_ were constructed first by amplifying the operon *mipBA* and the gene *mipA*, respectively, by PCR and cloned by sequence- and ligation-free cloning (2) into the pET15b-VP vector (S. Lory lab). Of note, the pET15b-VP is a vector designed for expression in both *E. coli* and *Pseudomonas* sp., thanks to two specific *ori* sites. During the PCR reaction, we added the sequence for six histidine residues at the end of the *mipA* gene. Then the pET15b-VP-*mipB/mipA-*His_6_ was mutated in order to add a Strep-tag at the C-terminus of MipB; the Strep-tag sequence was optimized using *Pseudomonas* codon usage (TGGAGCCACCCGCAGTTCGAAAAG). The resulting vector was verified by sequencing. All primers are listed in Table S3.

For gene deletion, allelic exchange vectors were designed with upstream and downstream flanking regions of approximately 500 bp and cloned into a pUC57 (ampicillin [Amp]) with *Hin*dIII/*Eco*RI, *Eco*RI/*Bam*HI, or *Hind*III/*Bam*HI restriction enzymes (Genewiz). Fragments for *mipA*, *mipBA*, and *parRS* deletions and *mipB*_3× FLAG_ C-terminal tagging were sub-cloned into pEXG2 (Gm). The allelic exchange vectors were conjugated into *P. aeruginosa* by triparental mating using pRK600 as a helper plasmid. Clones resulting from homologous recombination were selected on irgasan-containing LB plates and streaked onto NaCl-free LB plates with 10% sucrose (wt/vol) to select for plasmid loss. Sucrose-resistant mutants were verified for the gene deletion by PCR after verification of antibiotic sensitivity.

### Phylogenetic analysis

*P. aeruginosa* complete genomes (144) were retrieved from the Refseq database on the NCBI platform. Altogether, the database included 8 genomes from phylogenetic group 1, 7 from group 2, 68 from group 4, 38 from group 5, and 23 from group 3. This database was remodeled with the addition of clinical strains sequenced by the French National Reference Center for Antibiotic Resistance, Besançon. Sequences of interest, such as genes encoding the type III secretion system, as well as genes belonging to the *arn* and *mipAB* operons, were searched for in the genomes using the Sequence Extractor plugin in the BioNumerics software. Distances between genomes were established using Mash v.2.3 (83), and a phylogeny was generated with mashtree v.1.2.0 (84). To estimate the similarity between two genomes, the Mash software employs the MinHash technique. For each genome, multiple hash functions were applied to generate hash values. The set of these hash values forms the sketch (default set to 1,000). The size of *k*-mers used for MinHash calculations has been set by default to 21 *k*-mers, corresponding to the length of sequences hashed into MinHash values. These values were utilized to create a distinctive MinHash signature for each genome. Mashtree uses any common sequence file and calls the neighbor-joining algorithm, which is implemented in the software QuickTree (85).

Search for genes of interest was performed using the Sequence Extractor plugin of BioNumerics v.7.6.1 (Biomérieux) with at least 80% homology and 90% coverage to reference genes.

### Susceptibility testing

The MICs of selected antibiotics including PME (colistin sulfate, Sigma-Aldrich) were determined by broth microdilution method in Mueller-Hinton broth (MHB, Becton Dickinson) with adjusted concentrations of Mg^2+^ (from 10 to 12 µg/mL) and Ca^2+^ (from 20 to 25 µg/mL) in agreement with CLSI recommendations (86).

Isolation of one-step mutants with stable PME/colistin resistance was performed by plating 100 µL aliquots of log phase *P. aeruginosa* PAO1, PA14, PA7, and IHMA87 cultures (*A*_600_ equal to 1) on Mueller-Hinton agar (MHA) supplemented with 4–64 µg/mL of PME. From overnight culture, bacterial suspension was calibrated to an absorbance equal to 0.1 (*A*_600_) and diluted 1/10 to reach approximately 10^7^ CFU/mL. The suspension was grown in MHB medium to a final *A*_600_ of 1. One hundred microliters of the latter suspension was inoculated onto MHA plates (serial dilutions) and MHA plates containing increasing concentrations of PME (4–64 µg/mL), using the EasySpiral spiral plater system (VWR). After overnight growth, mutant frequency was calculated by dividing the number of PME-resistant mutants by the number of bacteria obtained on polymyxin-free MHA agar. Three independent experiments were carried out for each of the conditions studied.

#### Drug bactericidal activity

Overnight cultures of PAO1, PA14, PA7, and IHMA87 were diluted into fresh prewarmed MHB to yield an absorbance of *A*_600_ = 0.5 ± 0.05. The bacteria were incubated with a constant shaking (250 rpm) at 35°C for 30 min prior to the addition of PME at a final concentration ranging from 0.5 to 16.0 µg/mL. Fifty microliters of the culture was transferred in a sterile tube at a selected time point and inoculated on MH agar plate using easySpiral Spiral plater system (VWR). The survivors were counted after an overnight culture. Results were expressed as means of at least three independent experiments.

#### Stepwise adaptation of *P. aeruginosa* to PME

Ten microliters of an overnight culture was transferred into 10 mL of preheated MHB broth and incubated at 35°C with agitation at 250 rpm until reaching an *A*_600_ equal to 1. PME was added at a sub-inhibitory concentration of half the MIC. The bacterial suspension was incubated for 12 h at 35°C with agitation at 250 rpm. Strains showing bacterial growth were centrifuged twice at 3,500 × *g* and resuspended in MHB medium before being exposed to a PME concentration of 1 x MIC for 12 h. Sequential exposures were carried out up to 64× MIC (64 µg/mL). To determine the number of survivors for each strain studied, 50 µL of bacterial suspension was taken after 12 h of exposure to PME and plated on MHA media using the EasySpiral spiral plater system (VWR).

### ParR purification

From genomic DNA of PAO1 reference strain, a fragment of 705 bp corresponding to the complete coding sequence of the gene *parR* without the TGA stop codon, replaced by *Xho*I restriction site, was amplified with specific primers PR1f (5′-GGTGAATTCATGGACTGCCCTA-3′) and PR1r (5′-CTCCTCGAGGAGCTCCCAGCCCAG-3′). It was subsequently cloned into the pET28(a) vector using the restriction enzymes *Eco*RI and *Xho*I to generate the plasmid pET98 (pET28ΩparR). The pET98 recombinant plasmid was then transformed into *E. coli* BL21λDE3/pREP4 (87) and transformants were selected on ampicillin (100 µg/mL). The transformants were grown in 1 L of LB medium with constant shaking at 110 rpm at 30°C until an *A*_600_ = 0.8 before adding 1 mM of isopropyl β-D-1-thiogalactopyranoside (IPTG) (for 4 h) to induce the overproduction of the ParR protein. After centrifugation and sonication of the bacterial pellet in ice, crude protein extract was loaded on 1-mL His trap fast-flow column (GE Healthcare) equilibrated with buffer A (50 mM NaH_2_PO_4_, pH 7.5, 300 mM NaCl, 30 mM imidazole), and the protein was eluted with an imidazole gradient (300–500 mM) using the AKTA prime chromatography system (GE Healthcare). Fractions containing pure ParR protein (26.5 kDa) after visualization on a Coomasie gel (14%) were pooled and dialyzed against buffer B (50 mM NaH_2_PO_4_, pH 7.5, 300 mM NaCl, 50% glycerol) prior to determining protein concentration using the Bio-Rad Protein Assay.

### EMSA

From the genomic DNA of strain PAO1, the intergenic region (103 bp) of the *parR* and *mipB* genes containing the promoting region of gene *mipBA* (*P_mipBA_*) was labeled by PCR amplification using a combination of unlabeled primer with a primer end-labeled (625 nM) PM (5′-GACCCCGTTGACAGCG-3′) and PMrev (5′-TGGAACACCTGGCGGAAA-3′) with T4 polynucleotide kinase (0.075 U/µL) (New England Biolabs) and [γ-^32^P]-ATP (3,000 Ci/mmol) (PerkinElmer). The amplification was carried out in a 50 µL volume, and the products were purified as previously described (88). To phosphorylate the ParR protein, 150 µM of ParR protein was incubated in 20 µL of buffer C (50 mM Tris-HCL, pH 7.8, 20 mM MgCl_2_, 0.1 mM dithiothreitol) containing 178 pmol of acetyl [^32^P] phosphate (Hartmann Analytical) at 30°C for 1.30 h (ParR-P). The reaction mix was loaded onto a Sephadex G-50 spin column equilibrated with buffer C to remove excess of acetyl [^32^P] phosphate. The purified *P_mipBA_* labeled probes were incubated with various concentrations of purified ParR unphosphorylated (ParR) and phosphorylated (ParR-P) at 30°C for 20 min in 20 µL of buffer C. Then, the mixture was loaded with the DNA dye solution (40% glycerol, 0.025% bromophenol blue, and 0.025 xylene cyanol) on a 7.5% polyacrylamide gel. The gels were dried and analyzed by autoradiography.

### β-Galactosidase assays

Bacteria were grown to mid-exponential phase and PMB (0.25 µg/mL unless stated otherwise) and further grown for 90 min. β-Galactosidase activity was assessed according to (89) as described in (90). Briefly, 500 µL of bacteria was permeabilized by the addition of 20 µL of 0.1% SDS and 20 µL of chloroform and vortexed for 1 min. One hundred microliters of permeabilized bacterial suspension was added to 900 µL of Z buffer (0.06 M Na_2_HPO_4_, 0.04 M NaH_2_PO_4_, 0.01 M KCl, 1 M MgSO_4_, pH 7) supplemented with β-mercaptoethanol (0.27% [vol/vol]) and incubated at 28°C. Enzymatic reaction was started by the addition of 200 µL of *ortho*-nitrophenyl-β-galactoside (4 mg/mL) and stopped with 500 µL of 1 M Na_2_CO_3_ solution. Absorbance at 420 nm was read using a spectrophotometer after sedimentation of cell debris. Promoter activities are expressed in Miller units [(*A*_420_ × 1,000)(*t*_min_ × vol_mL_ × A_600_)]. Experiments were performed in three biological replicates.

### Genetic environment visualization

Genomic DNA sequence spanning from *algA* to *fruA* genes (for the study of *arn* operon) and downstream of *parR* (for the study of *mipBA*) of the different *P. aeruginosa* strains were retrieved from Pseudomonas.com (91) and visualized using Clinker tool (23).

### Protein predictions and genetic neighbor analysis

Signal peptides were predicted using SignalP v.5.0 (45). The three dimensional structure predictions of MipB and MipA/MipB complex were performed using AlphaFold (37) and AlphaFold-Multimer (46). Pymol was used to calculate electrostatic surface potential and generate figures.

WebFlaG was used to study genetic neighbors of *mipA* (76). MipA FASTA sequence from MipA_IHMA87_ was imputed, and a list of 50 homologs was searched by BlastP in the Atkinson Lab reduced database.

### Bacterial fractionation

Bacterial fractionation was performed according to the protocol described in reference 92. Briefly, bacteria were grown to exponential phase (*A*_600_ of 1). A total amount of 10^10^ bacteria were pelleted at 4°C and resuspended in 1 mL of buffer A (20 mM Tris-HCl, pH 8, 200 mM MgCl_2_) supplemented with protease inhibitor cocktail (PIC, Roche) and lysozyme to a final concentration of 0.5 mg/mL. The sample was incubated for 30 min at 4°C on a rotating wheel. Periplasmic fraction was recovered after centrifugation at 11,000 × *g* for 15 min at 4°C. The spheroplast-containing pellet was washed with 1 mL of buffer B (20 mM Tris-HCl, pH 8, 20% sucrose) supplemented with PIC and resuspended in 1 mL of buffer B before a sonication step (5 min, 40% intensity, 10 s on/10 s off) on ice. Bacteria debris were removed by centrifugation for 15 min at 8,000 × *g* at 4°C, and the supernatant, composed of bacterial cytosol and membranes, was further centrifuged for 10 min at 8,000 × *g* at 4°C. Cytosolic and membranes components were separated by ultracentrifugation at 200,000 × *g* (Rotor TLA120 Beckman) for 45 min at 4°C. The supernatant containing the cytosol was recovered; the pellet was washed twice with 1 mL of buffer C (20 mM Tris-HCl pH 8.0, 20 mM MgCl_2_) supplemented with PIC. Bacterial membranes were resuspended in 500 µL of buffer B using a potter. All fractions were resuspended or diluted in 4× SDS-PAGE loading buffer and boiled for 10 min prior to SDS-PAGE or Western blot analyses.

### Inner- and outer-membrane separation

Inner and outer membranes were separated using a discontinuous sucrose gradient as described in reference (55). In brief, bacteria were grown to exponential phase, and 2.5 × 10^11^ bacteria were pelleted for 15 min at 4°C and resuspended in 25 mL of buffer A (10 mM Tris-HCl pH 7.4, RNase 10 µg/mL, DNase 10 µg/mL, and 20% sucrose) supplemented with PIC. Bacteria were lysed by sonication at 50% intensity, 7 min, 30 s on/ 30 s off), and remaining cell debris were removed by a centrifugation step. Supernatants were ultracentrifuged at 200,000 × *g* for 45 min at 4°C (TI45 Beckman rotor). Total membrane pellet was resuspended in 500 µL of buffer B (10 mM Tris-HCl, pH 7.4, 5 mM EDTA, 20% sucrose, and PIC) and loaded onto an eight-1.5 mL-layer sucrose gradient (from bottom to top) with sucrose concentrations of 55%, 50%, 45%, 40%, 35%, and 30% and submitted to another ultracentrifugation step of 72 h at 90,000 × *g* (Beckman SW41 swinging rotor) at 4°C. Finally, 500 µL fractions were collected and further characterized by SDS-PAGE and Western blot analyses.

### Western blot analyses

Sample protein content present on the SDS-PAGE was transferred onto a polyvinylidene difluoride membrane (GE Healthcare) for 90 min (25 V, 125 mA) in Laemmli buffer with 20% ethanol. Membranes were blocked for 1 h at room temperature in 5% (wt/vol) dry milk in phosphate-buffered saline (PBS)-Tween 0.1% and labeled with primary antibodies for 1 h. Primary antibodies were used at the following concentrations: anti-MipA (1/2,000, rabbit; Biotem), anti-DsbA (1/10,000; obtained from R. Voulhoux, CNRS, Marseille, France), anti-EF-Tu (1/10,000, mouse; Hycult Biotech), anti-XcpY (1/2,000, rabbit [93]), anti-FLAG (1/2,000, mouse; Sigma F3165), anti-OprM (1/2,000, rabbit; given by P. Plésiat, Besançon), anti-RpoA (1/5,000, mouse; BioLegend 663104), anti-Strep-tag (1/6,000, mouse) and anti-His_6_ (1/4,000–6,000, mouse). The secondary horseradish peroxidase (HRP)-conjugated antibodies directed against rabbit or mouse were used at a 1/20,000–50,000 dilution (Sigma). Detection of luminescent signal was performed with a Luminata Western HRP substrate kit (Millipore). Polyclonal rabbit antibodies were raised against purified MipA-His_6_ following the manufacturer recommendations (Biotem).

### MipB and MipA co-purification

*E. coli* BL21(DE3)RIL containing the pET15b-VP-*mipB*-S*trep*/*mipA-His*_6_ plasmid was grown in LB (1 L) with appropriate antibiotic concentrations at 37°C with shaking. When bacteria reached an *A*_600_ of 1.15, protein expression was induced with 1 mM IPTG and further grown for 2.5 h at 37°C with shaking. Bacterial culture was then centrifuged at 5,000 × *g*, for 20 min at 4°C, and the pellet was resuspended in 100 mL of lysis buffer (25 mM Tris-HCl, pH 7.5, 150 mM NaCl, 10% glycerol [vol/vol], 1 mM EDTA, and 2% N-lauroylsarcosine [wt/vol]) supplemented with protease inhibitor cocktail (cOmplete ULTRA Tablets, Roche) and DNAseI (10 µg/mL). Bacterial cells were lysed with a M110-P microfluidizer (Microfluidics) with six passages at 18,000 psi and centrifuged at 39,000 × *g* for 45 min at 4°C to remove bacterial debris. The soluble fraction was loaded onto a StrepTrap HP 1 mL affinity column (GE Healthcare) at 4°C, previously equilibrated with 25 mM Tris-HCl, pH 8.0, 150 mM NaCl, 1 mM EDTA, and 2% N-lauroylsarcosine (wt/vol). The column was washed with five-column volumes of buffer before elution with the same buffer containing 2.5 mM of desthiobiotin (Sigma D1411). Eluted fractions were analyzed by 12%-15% SDS-PAGE and immunoblotting with anti-His and anti-MipA for MipA and anti-Strep for MipB.

### MipA purification

Freshly transformed *E. coli* BL21(DE3)C41 colonies harboring pET15b-VP-*mipA*-His_6_ were grown at 30°C overnight in LB with 100 µg/mL Amp with shaking. The next day, culture was diluted in 1 L LB-Amp and grown at 37°C until an *A*_600_ of 1.0, then protein expression was induced with 0.5 mM IPTG and further grown for 2.5 h at 37°C with shaking. Bacterial culture was then centrifuged at 6,000 × *g* for 30 min at 4°C, and the pellet was resuspended in 200 mL of lysis buffer (25 mM Tris-HCl, pH 8.0, 150 mM NaCl, 10% glycerol [vol/vol], 2% N-lauroylsarcosine [wt/vol]) supplemented with protease inhibitor cocktail (cOmplete ULTRA Tablets, Roche), DNaseI (1 µg/mL), and RNaseI (10 µg/mL). Bacterial cells were lysed with a M110-P microfluidizer (Microfluidics) with 10 passages at 15,000 psi and centrifuged at 39,000 × *g* for 1 h at 4°C to remove bacterial debris. The soluble fraction was loaded at low speed (0.2 mL/min) at 4°C onto a HisTrap-HP 1 mL affinity column (GE Healthcare) equilibrated with buffer A (20 mM NaPi, pH 7.7, 500 mM NaCL, 20 mM imidazole, and 1% N-lauroylsarcosine). The column was washed with 40 volumes of buffer A before elution in with the same buffer A containing 175 mM imidazole. Dithiothreitol (DTT; 10 mM) and EDTA (5 mM) were added to the fractions containing MipA-His_6_ to avoid disulfide bridge formation and inhibit metalloproteases, respectively. The protein samples were then injected onto a Superdex200 Increase 10/300 Gl (GE Healthcare) equilibrated with 25 mM HEPES, pH 7.5, 150 mM NaCl, 1 mM EDTA, and 0.1% LAPAO (3-dodecylamido-N,N′-dimethylpropyl amine oxide) (wt/vol). MipA-His_6_, eluted in the peak corresponding to an elution volume of 12 mL, was used for binding assay. Protein purity was analyzed by 15% SDS-PAGE, and protein concentration was determined by *A*_280_.

### Protein stability measurement using nano-DSF

Protein stability and binding affinity were determined using nano-DSF that allows the use of dye-free samples containing detergent. Samples (purified MipA_His6_ and selected ligands) were filled into capillaries and loaded on the Prometheus NT48 machine (Nanotemper). Temperature was increased by 0.5°C or 1°C/min from 20°C to 95°C, and fluorescence at 330 nm (F330) and 350 nm (F350) was recorded. The ratio (F350:F330) was calculated and plotted. The inflection point corresponding to a maximum in the first derivative (slope) of F350:F330 gives the value of the Tm. For binding affinities, purified MipA_His6_ (5 µM) was incubated for 2 h at room temperature with PMB or PME ranging from 76 nM to up to 2.5 mM, then analyzed by nano-DSF. Data were analyzed using FoldAffinity online tool (60, 61). Only the signals between 40°C and 70°C were used for the first fluorescence fitting step using the “global-CP” model, then isothermal analysis were done around unfolding temperature (Tm = 52°C). Finally, the unfolded protein fraction versus ligand concentration was fitted using a one site model to obtain a *K*_D_. Only the best fittings were taken into consideration at three fixed temperatures: 51°C, 52°C, and 53°C. Three negatively charged molecules were used as negative controls in nano-DSF: two AMPs, magainin II (SIGMA) and indolicidin (Thermo Scientific), and the antibiotic streptomycin (Sigma). The *K*_D_ error was estimated using the asymptotic method.

### MS-based quantitative proteomic analysis of bacterial membranes

Bacterial cultures (30 mL LB) were inoculated at an *A*_600_ of 0.1 and grown to *A*_600_ of ~0.6. PMB was added when needed (0.25 µg/mL) for 90 min. Bacteria were harvested and resuspended in 1 mL of buffer B (same buffers as in bacterial fractionation) supplemented with PIC. Bacteria were lyzed by sonication for 5 min (40% intensity, 10 s on/10 s off), and bacterial debris were eliminated by a centrifugation step of 15 min at 6,000 × *g* at 4°C. The supernatant was ultracentrifuged at 200,000 × *g* for 45 min at 4°C, and the soluble fraction was removed. Membranes pellets were washed and resuspended in 100 µL of buffer C. Loading buffer was added to the samples, which were then boiled for 10 min. Presence of MipA and MipB in each sample was assessed by Western blot analysis.

Three replicates of membrane fraction were prepared for each analyzed strain. The proteins were solubilized in a Laemmli buffer and stacked on top of a 4%–12% NuPAGE gel (Invitrogen). After staining with R-250 Coomassie Blue (BioRad), proteins were digested in gel using modified trypsin (sequencing purity, Promega), as previously described (55). The resulting peptides were analyzed by online nanoliquid chromatography coupled to tandem mass spectrometry (MS/MS) (Ultimate 3000 RSLCnano and Q-Exactive HF, Thermo Fisher Scientific) using a 180-min gradient. For this purpose, the peptides were sampled on a precolumn (300 µm × 5 mm PepMap C18, Thermo Scientific) and separated in a 75 µm × 250 mm C18 column (Reprosil-Pur 120 C18-AQ, 1.9 µm; Dr. Maisch). The MS and MS/MS data were acquired using Xcalibur (Thermo Fisher Scientific).

Peptides and proteins were identified by Mascot (Matrix Science) through concomitant searches against the NCBI database (*P. aeruginosa* strain: IHMA879472 taxonomy, March 2021 download) and a homemade database containing the sequences of classical contaminant proteins found in proteomic analyses (human keratins, trypsin, etc). Trypsin/P was chosen as the enzyme, and three missed cleavages were allowed. Precursor and fragment mass error tolerances were set at 10 and 20 ppm, respectively. Peptide modifications allowed during the search were carbamidomethyl (C, fixed), acetyl (protein N-term, variable), and oxidation (M, variable). Proline software v.2.2.0 (94) was used for the compilation, grouping, and filtering of the results (conservation of rank 1 peptides, peptide length ≥6 amino acids, false discovery rate [FDR] of peptide-spectrum match identifications <1% [95], and minimum of one specific peptide per protein group). Proline was then used to perform a MS1 label-free quantification of the identified protein groups based on razor and specific peptides.

Statistical analysis was then performed using ProStaR software v.1.30.5 (96). Proteins identified in the contaminant database, proteins identified by MS/MS in less than two replicates of one condition, and proteins detected in less than three replicates of one condition were discarded. After log_2_ transformation, abundance values were normalized by the variance stabilizing normalization method before missing value imputation (slsa algorithm for partially observed values in the condition and DetQuantile algorithm for totally absent values in the condition). Statistical testing was then conducted using Limma, whereby differentially expressed proteins were sorted out using a log_2_ (fold change) cutoff of 1 and a *P* value cutoff of 0.01, leading to FDRs inferior to 3% according to the Benjamini-Hochberg estimator. Proteins found differentially abundant but identified by MS/MS in less than two replicates and detected in less than three replicates in the condition in which they were found to be more abundant were manually invalidated (*P* value = 1).

### RT-qPCR

After total RNA extraction from 2 mL of bacterial exponential cultures grown to *A*_600_ of 1 using TRIzol Plus RNA Purification Kit and TURBO DNA-Free kit (Invitrogen) treatment, cDNA synthesis was carried out with the SuperScript IV Reverse Transcriptase (Invitrogen). Quantification is based on real-time SYBR green amplification molecules with specific target primers (Table S3) using Luna Universal qPCR Master Mix (Biolabs). The genomic DNA absence was verified by including samples without reverse transcriptase. Relative mRNA expression is calculated using the CFX Manager software (BioRad), using *rpoD* as reference. Statistical analyses were performed with GraphPad Prism v.9. The sequences of primers are listed in Table S3.

### Statistical analysis

Data were statistically analyzed using GraphPad Prism v.9. For multiple comparisons, one-way analysis of variance or Kruskall-Wallis test was performed, depending on the data normality (Shapiro test), followed by a Tukey or a Dunn test for normally and non-normally distributed data sets, respectively. To compare two groups, a Student *t*-test or a Mann-Whitney test was applied, depending on the normality of the data. *P < 0.05, **P < 0.01, ***P < 0.001, ****P < 0.0001.

## Data Availability

MS data have been deposited to the ProteomeXchange Consortium via the PRIDE partner repository (97) with the data set identifier PXD043253. Analyzed data are available in Table S1.

## SUPPLEMENTAL MATERIAL

Supplemental material mbio.02211-23-s0001.docx Supplemental figures and Table S2.

Table S1 mbio.02211-23-s0002.xlsx Proteomic analysis of bacterial membranes upon polymyxin treatment.

ASM does not own the copyrights to Supplemental Material that may be linked to, or accessed through, an article. The authors have granted ASM a non-exclusive, world-wide license to publish the Supplemental Material files. Please contact the corresponding author directly for reuse.
