# Supplementary material for: Pseudomonas aeruginosa MipA-MipB envelope proteins act as new sensors of polymyxins
Source: mBio. 2024 Feb 12;15(3):e02211-23. doi: 10.1128/mbio.02211-23 (PMC10936184; doi:10.1128/mbio.02211-23)
Supplement: Supplemental material — Supplemental figures and Table S2. [file mbio.02211-23-s0001.docx]

***Pseudomonas aeruginosa* MipA-MipB envelope proteins act as new sensors of polymyxins**

Manon Janet-Maitre^1*#^, Viviana Job^1^, Maxime Bour^2,3^, Mylène Robert-Genthon^1^, Sabine Brugière^4^, Pauline Triponney^3^, David Cobessi^5^, Yohann Couté^4^, Katy Jeannot^2,3,6^ and Ina Attrée^1#^

**Supplemental information**


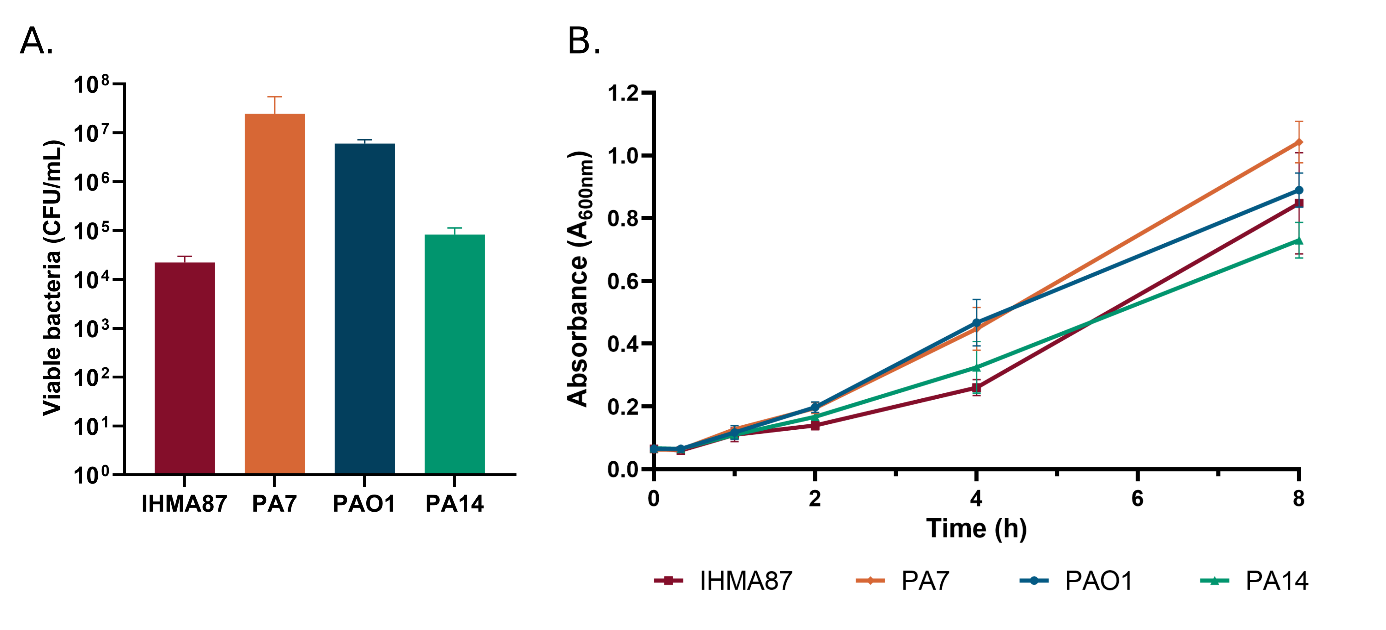


**Figure S1. *Arn* negative strains are capable of adaptation to polymyxins. A.** Bacterial tolerance to PME. Number of viable bacteria at 64 µg/mL of PME (*n=3*) **B.** Bacterial growth curve. Strains were grown and absorbance at 600nm was measured at different timepoints (*n=3*).

**
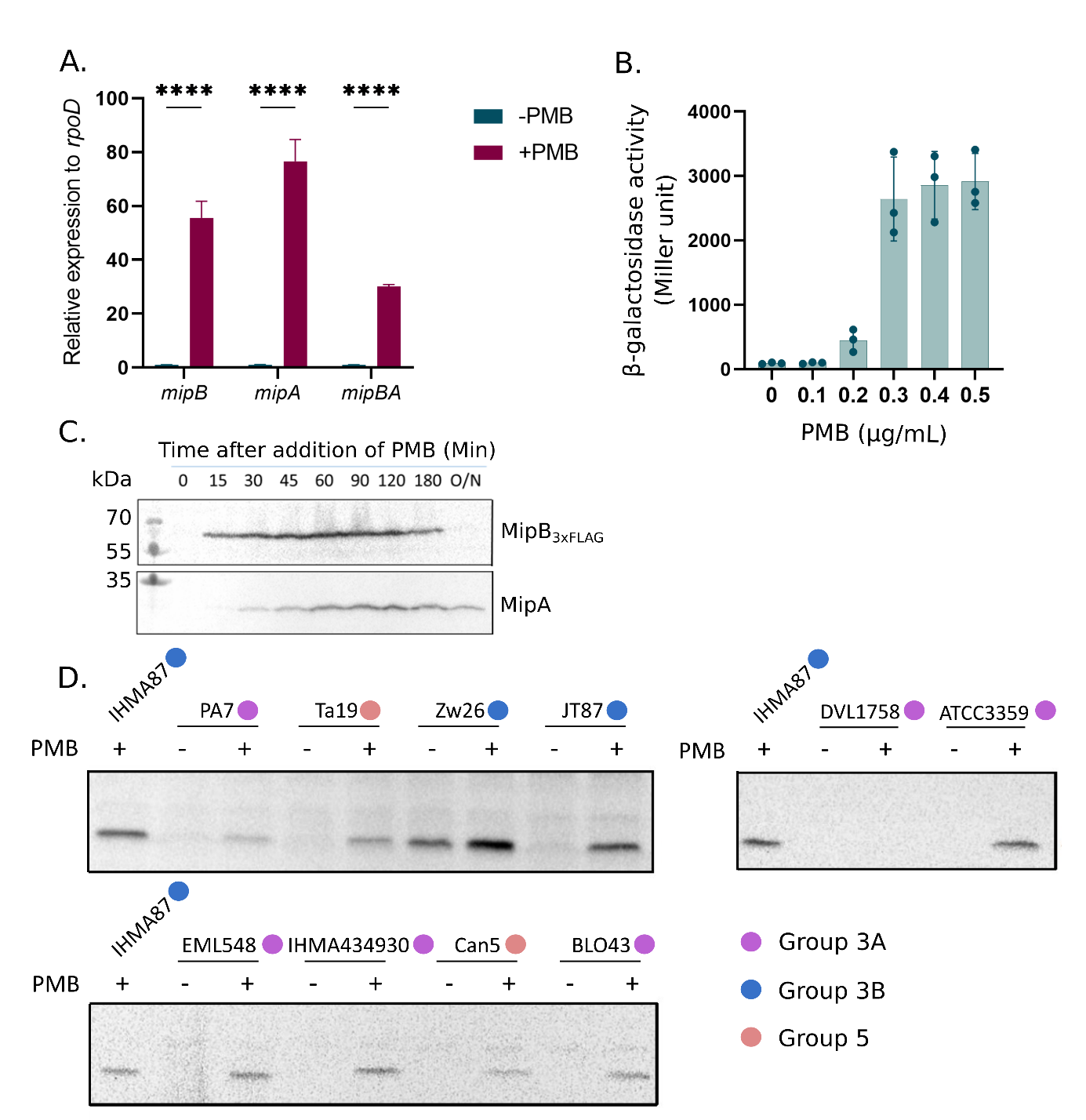
**

**Figure S2. *mipBA* induction upon sub-lethal PMB treatment is rapid, dose-dependent and conserved among *P. aeruginosa* strains. A.** *mipBA* operon is induced in response to sub-lethal concentration of PMB measured by RT-qPCR (*n=3*). **B.** Dose-response of *P_mipBA_* promoter activity to PMB performed by β-galactosidase assay (*n=3*). **C.** Kinetics of MipB and MipA synthesis upon PMB sub-lethal treatment (0.25 µg/mL) in IHMA87 *mipB_3xFLAG_* analyzed by immunoblot using anti-FLAG and anti-MipA antibodies.**D.** The induction of MipA in response to PMB is conserved across strains from group 3A, groups 3B and 5 in *P. aeruginosa* species.


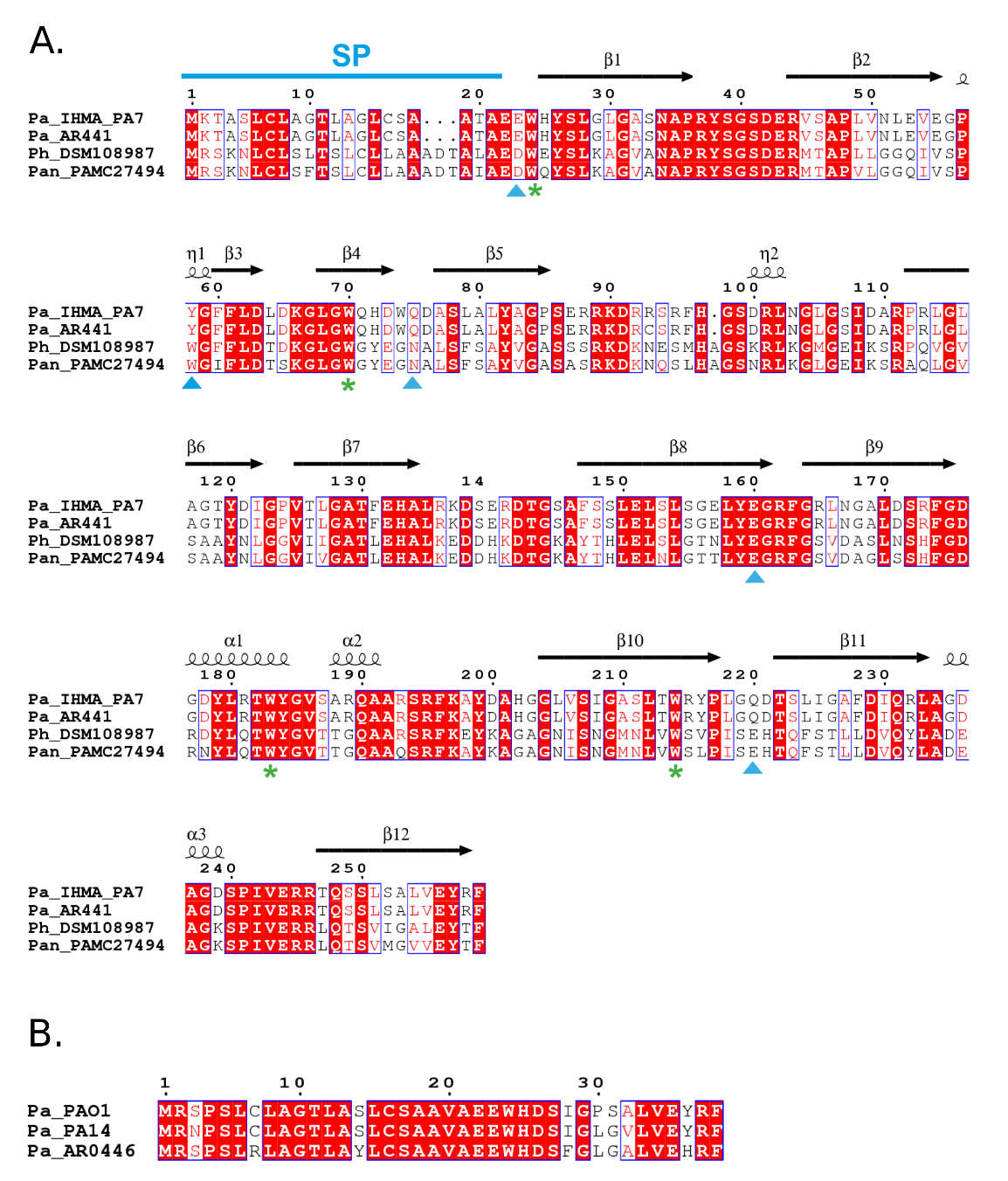


**Figure S3. Sequence alignment of MipA. A.** Proteins homologous to MipA from *P. aeruginosa* IHMA87 were aligned using CLUSTAL. The secondary structure of MipA-IHMA87 retrieved from the model is shown above the sequence alignment. Strains used for the alignment were *P. aeruginosa* IHMA87, PA7, AR441 and CR1, and *P. heamolytica* DSM108987 and *P. antartica* PAMC 27494. Full and weakly conserved residues are shaded in red box and blue box, respectively. The four-conserved Trp residues important for the OM insertion are highlighted by an asterisk, the residues putatively important for interaction with MipB are depicted by a cyan arrow. Signal peptide (SP). The alignment was visualized by ESPript 3 [1]. **B.** Sequence alignment of short versions of the MipA protein from PAO1, PA14 and AR0446.


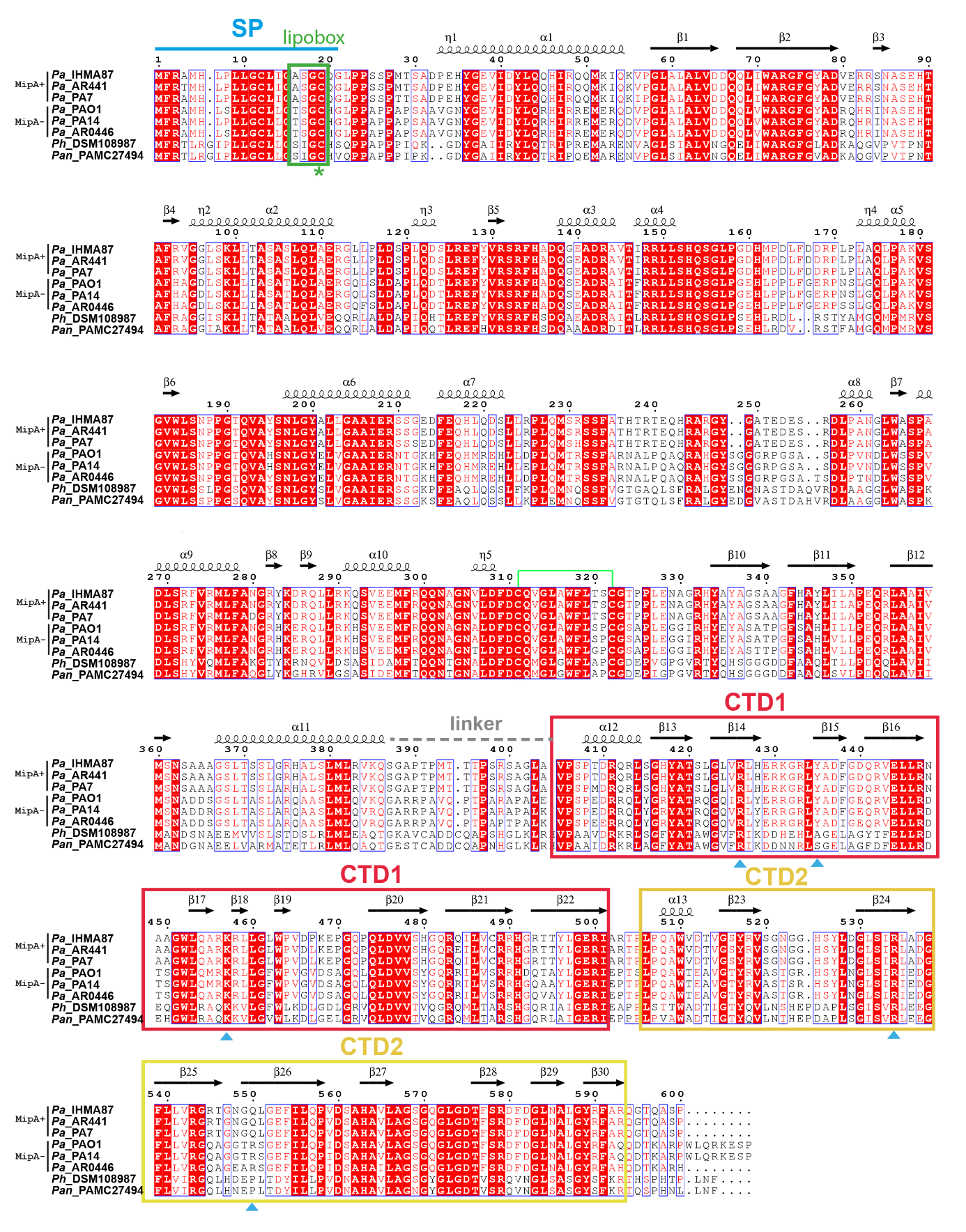
**Figure S4. Sequence alignment of MipB proteins.** Proteins homologous to MipB from *P. aeruginosa* IHMA87 were aligned using CLUSTAL. The secondary structure of MipB-IHMA87 retrieved from the model is shown above the sequence alignment. The putative disulfide bridge between Cys311 and Cys321 is depicted with the green line. Note the divergent sequence between MipA+ and MipA-negative strains within the *P. aeruginosa* group.  Strains used for alignment were IHMA87, AR441 and PA7 which contain MipA and PAO1, PA7 and AR0446 which only have the truncated version of MipA*. The MipB sequences of *P. heamolytica* strain DSM108987 and *P. antartica* PAMC 27494 were included in the alignment. Full and weakly conserved residues are shaded in red and blue boxes, respectively. MipB has a predicted lipobox (green box) with one Cys (asterisk). The predicted signal peptide (SP), the large beta-lactamase like domain followed by the linker loop (dashed gray line) and the two C-terminal domains (CTD1 and CTD2) are shown. Note the high conservation of the CTDs inside the MipA+ and MipA- groups.  The residues putatively important for MipA interaction are highlighted by a cyan triangle. The alignment was visualized by ESPript 3 [1].


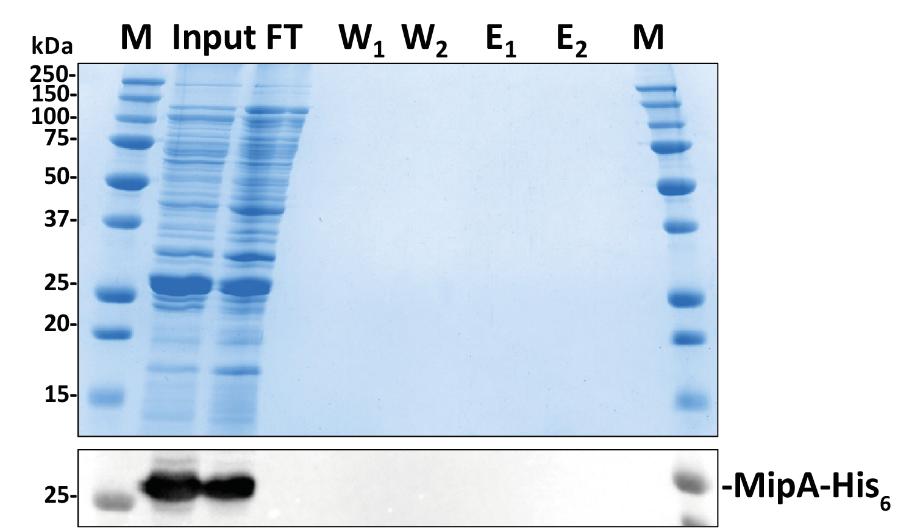


**Figure S5. MipA-His_6_ is not retained by the Strep column.** Lysate from C41 overexpressing MipA-His_6_ (Input) was loaded onto a Strep column. Samples were collected after washing and elution with a buffer containing 2.5 mM of desthiobiotin and analyzed by SDS-PAGE. Coomassie staining (top) and immunoblotting using anti-His antibody and anti-MipA (bottom) are presented above. FT: flowthrough, W: wash, E_1_-E_2_: elutions.

**
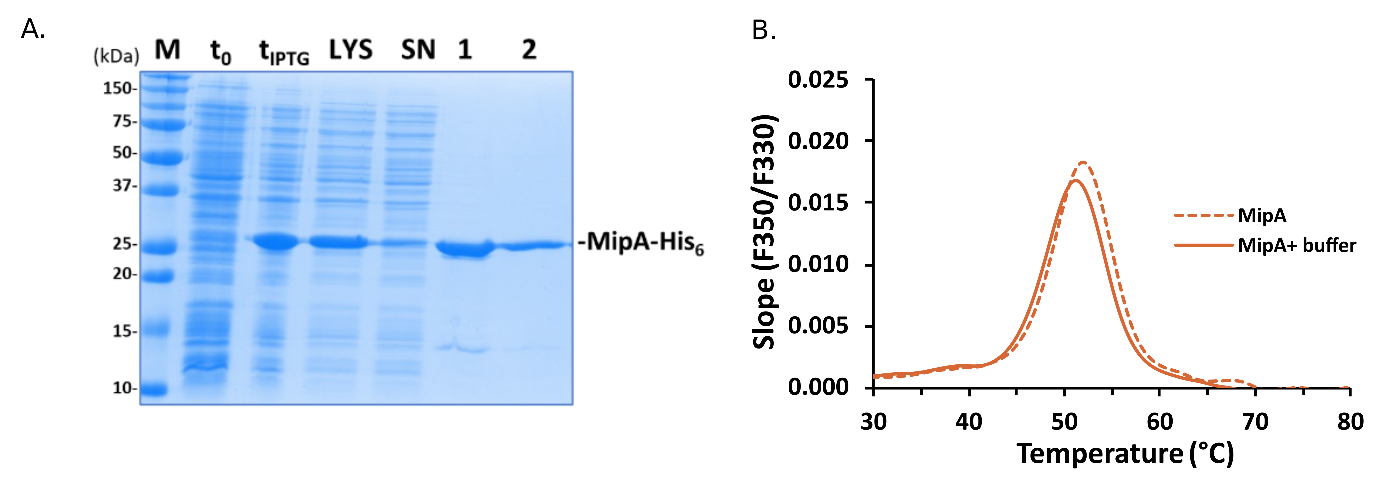
**

**Figure S6. The thermal stability of purified MipA is not modified by the buffer. A.** SDS-PAGE summarizing the purification steps of MipA-His_6_. Protein expression in *E. coli* C41 was induced in exponential phase (t0) by IPTG addition (t_IPTG_), bacteria were lysed (LYS) in presence of 2% N-lauroylsarcosine (w/v), the soluble proteins (SN) were loaded on an affinity His-Trap column and eluted with imidazole (**1**). MipA was further purified on Superedex200 (**2**) in presence of 0.1% LAPAO. **B.** Thermal stability of MipA is not modified by the addition of buffer. Pure MipA alone (in orange, dashed line) or incubated for 2h at RT with the buffer, was heated from 20 to 95 °C. Protein folding/unfolding was followed by tryptophan fluorescence at 330 and 350nm using nano-DSF. The slope of the ratio (F350/F330) is plotted at the different temperatures, the maximum corresponds to the melting temperature (Tm) of the protein.

**
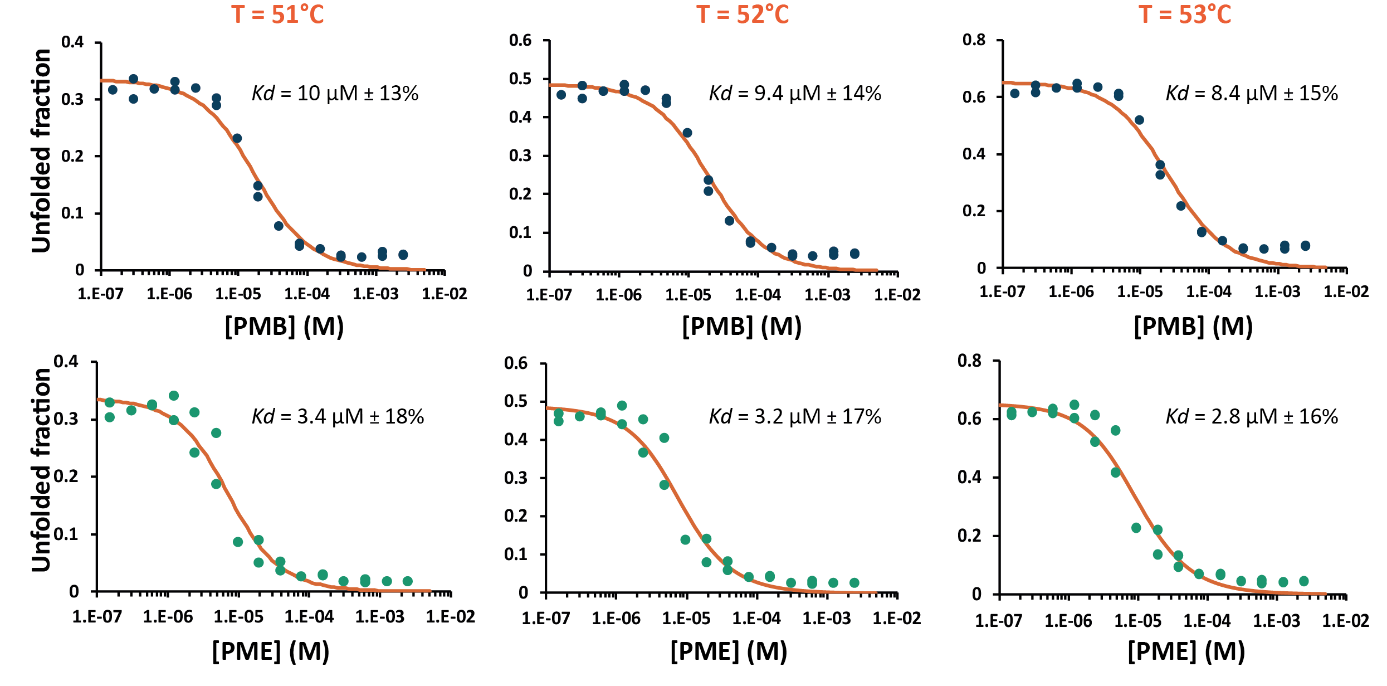
**

**Figure S7. Calculated K_D_ between MipA and PMB/PME.** MipA (5 µM) was mixed with increasing concentration of PMB (top panels) or PME (lower panels) and analyzed by nano-DSF. Isothermal analysis using online FoldAffinity tool allowed to determine K_D_ values at 51 °C, 52°C and 53°C, showing a specific interaction between MipA and PMB/PME [2,3]. Errors were estimated using the asymptotic method (*n=3*).

**
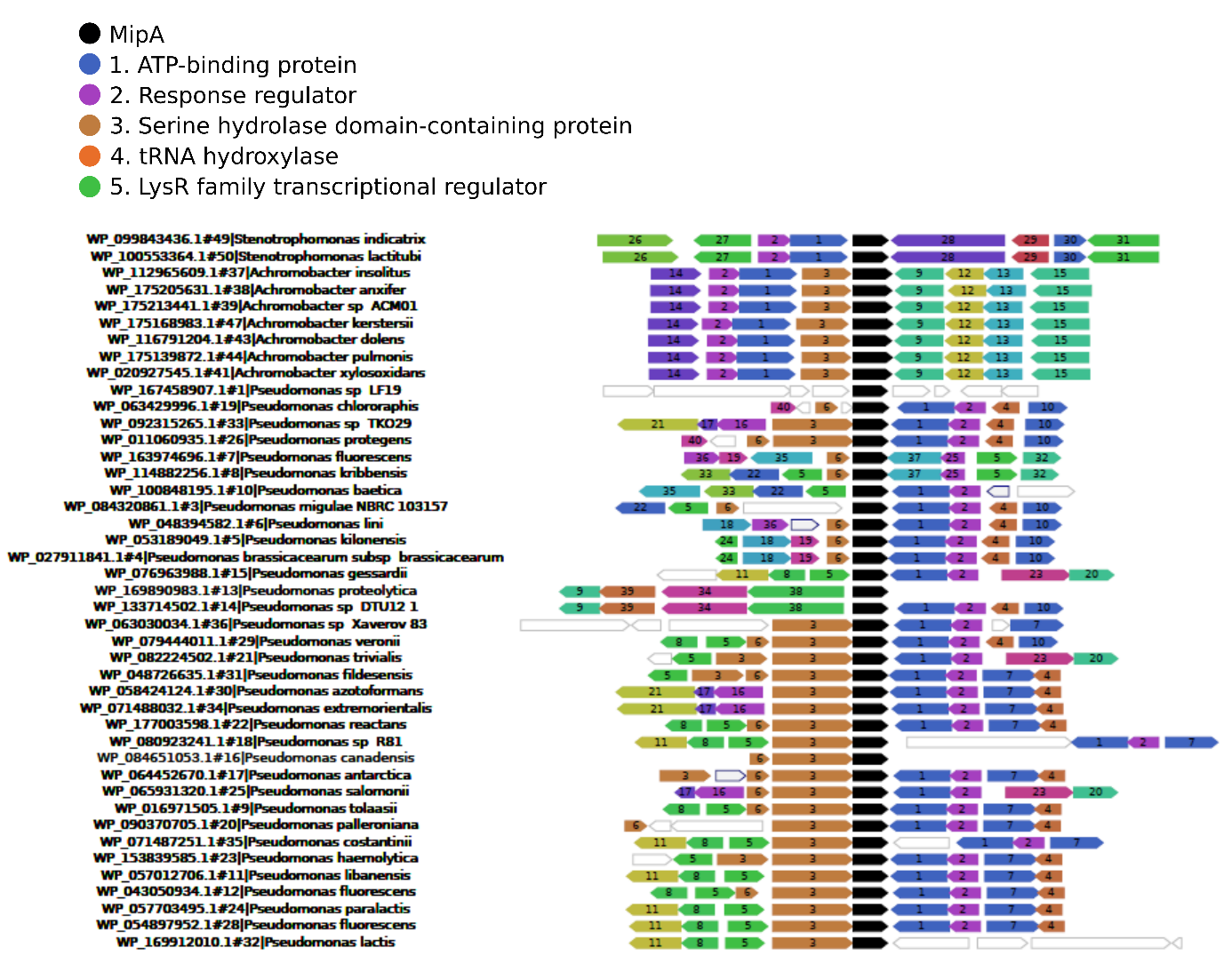
**

**Figure S8. *mipA* genetic neighborhood conservation.** 50 MipA protein homologs were retrieved by BLASP together with the corresponding neighboring genes within the Atkinson lab reduced database and most common genetic neighbors’ protein predictions are indicated. Analysis and image generated with WebFlaGs [4].

**Table S1. Data from proteomic analysis of bacterial membranes**

**Table S2. Bacterial strains and plasmids**

| **Bacteria** | **Features / Source** | **Reference/origin** |
| --- | --- | --- |
| ***Pseudomonas aeruginosa*** | |  |
| IHMA879472/  AZPAE15042 | Isolated from a urinary infection in Germany, group 3B | IHMA^1^ collection [5,6] |
| IHMA87Δ*mipA* | IHMA87 *mipA* deletion mutant | This work |
| IHMA87Δ*mipBA* | IHMA87 *mipBA* deletion mutant | This work |
| IHMA87Δ*parRS* | IHMA87 *parRS* deletion mutant | This work |
| IHMA87 *mipB*_3xFLAG_ | IHMA87 C-ter FLAG tagged MipB | This work |
| IHMA87::pminiCTX-*PmipBA*-lacZ | IHMA87 *with PmipBA-lacZ* transcriptional fusion (Tc^R^) | This work |
| PA7 | Isolated from a wound in Argentina, group 3A | [7] |
| PAO1 | Isolated from a wound isolated, group 1 | [8], Lab collection |
| PA14 | Isolated from a burnt infection, group 2 | [9] |
| EML548 | Isolate from Germany, group 3A | [10] |
| ATCC33359/ EML545 | Isolated from a water sample in Germany, group 3A | [10] |
| IHMA434930/ AZPAE14901 | Isolated from intra-abdominal tract infection in India, group 3A | IHMA^1^ collection [10] |
| BL043 | Isolated from a bacteremia in the USA, group 3A | [10] |
| Zw26 | Isolated from a cystic fibrosis sputum in Germany, group 3B | [10] |
| JT87 | Isolated from a urinary infection in the USA, group 3B | [10] |
| Ta19 | Isolated from a urine sample in Australia, group 5 | [10] |
| Can5 | Isolated from a dog infection in the United Kingdom, group 5 | [10] |
| DVL1758 | Isolated from a shallow pond in Belgium, group 5 | [10] |
| ***Escherichia coli*** |  |  |
| DH5α | Laboratory strain | Lab collection |
| TOP10 | Cloning strain | Invitrogen |
| BL21(DE3)RIL | *E. coli* expressing extra copy of tRNA genes for codone rare: *arg*U (AGA, AGG), *ile*Y (AUA) and *leu*W (CUA) (Cm^R^) | This work |
| BL21(DE3)C41 | *E. coli* for expression of MipA-His_6_ | This work |
| BL21(DE3)RIL pET15bVP-*mipB*-*strep*/*mipA*-His_6_ | *E. coli* for co-expression of MipA-His_6_ and MipB-Strep | This work |
| **Plasmids** |  |  |
| pRK600 | Helper plasmid with conjugative properties (Cm^R^) | [11] |
| pEXG2 | Allelic exchange vector (Gm^R^), *sacB* | [12] |
| pEXG2-mut-*mipBA* | pEXG2 carrying DNA fragment for *mipBA* deletion in IHMA87 (Gm^R^) | This work |
| pEXG2-mut-*parRS* | pEXG2 carrying DNA fragment for *parRS* deletion in IHMA87 (Gm^R^) | This work |
| pEXG2-mut-*mipB*_3xFLAG_ | pEXG2 carrying DNA fragment for C-ter FLAG tagging of MipB in IHMA87 (Gm^R^) | This work |
| pET15b-VP | Engeneered vector with two *ori* sites for expression of proteins in both *E. coli* and *P. aeruginosa* | S. Lory Lab |
| pET15b-VP-MipB/MipA-His_6_ | Vector with operon *mipAB* cloned into *Nco*I/*Bam*HI sites (Amp^R^) | This work |
| pET15b-VP-MipB-Strep/MipA-His_6_ | Vector used for co-purification of MipB-Strep and MipA-His_6_ (Amp^R^) | This work |
| pET15b-VP-MipA-His_6_ | Vector use for purification of MipA | This work |

^1^ International Health Management Association, USA

**Table S3. Primers**

| **Primers** | **Sequence (5’-3’)** | **Purpose** |
| --- | --- | --- |
| MipBStrep-rv | CcagggcacccaggcatcacccTGGAGCCACCCGCAGTTCGAAAAGtgaaccaatcgaaaggaatccctc | Used for mutagenesis (add of Strep tag sequence to Cter MipB) |
| MipBStrep-fw | gagggattcctttcgattggttcaCTTTTCGAACTGCGGGTGGCTCCAgggtgatgcctgggtgccctgG | Used for mutagenesis (add of Strep tag sequence to Cter MipB) |
| NcoI-MipB | tttaagaaggagatataccatgttccgcgcaatgcatctcc | Used for cloning *mipAB* operon by SLIC reaction in pET15b-VP |
| MipB-BamHI-His_6_ | gctttgttagcagccggatcc**tca**gtgatgatgatgatgatggggtgatgcctgggtgccc | Used for cloning *mipAB* operon by SLIC reaction in pET15b-VP |
| MipA-BamHI-His_6_ | gctttgttagcagccggatcctcagtgatgatgatgatgatggaagcggtattccaccagcgcg | Used for cloning *mipA* operon by SLIC reaction in pET15b-VP |
| NcoIMipA | tttaagaaggagatataccatgaaaaccgcctccctgtg | Used for cloning *mipA* operon by SLIC reaction in pET15b-VP |
| qPCR-mipA-F | tatccccttggccaggacac | Used for RT-qPCR |
| qPCR-mipA-R | cgagctttgcgtacgacgtt | Used for RT-qPCR |
| qPCR-mipB_F | gttcgccaacggccggtaca | Used for RT-qPCR |
| qPCR-mipB_R | aggacgttgccggcgttctg | Used for RT-qPCR |
| qPCR-mexX_F | cagaaccgcctgaagatcgt | Used for RT-qPCR |
| qPCR-mexX_R | gccgctttctccacgtagat | Used for RT-qPCR |
| qPCR-mexY_F | atgcacatccaatggaccgg | Used for RT-qPCR |
| qPCR-mexY_R | cagcccagcaggaataggg | Used for RT-qPCR |
| qPCR-oprA_F | ggcaacaacagttcaccgac | Used for RT-qPCR |
| qPCR-oprA_R | aggttgcggttgtgctcc | Used for RT-qPCR |
| qPCR-rpoD_F | ctgccggaggatatttcaga | Used for RT-qPCR |
| qPCR-rpoD_R | atacgttgatccccatgtcg | Used for RT-qPCR |

**References**

1. Gouet P, Robert X, Courcelle E. ESPript/ENDscript: Extracting and rendering sequence and 3D information from atomic structures of proteins. Nucleic Acids Res. 2003;31: 3320–3323. doi:10.1093/nar/gkg556

2. Niebling S, Burastero O, Bürgi J, Günther C, Defelipe LA, Sander S, et al. FoldAffinity: binding affinities from nDSF experiments. Sci Rep. 2021;11: 9572. doi:10.1038/s41598-021-88985-z

3. Burastero O, Niebling S, Defelipe LA, Günther C, Struve A, Garcia Alai MM. eSPC: an online data-analysis platform for molecular biophysics. Acta Crystallogr Sect Struct Biol. 2021;77: 1241–1250. doi:10.1107/S2059798321008998

4. Saha CK, Sanches Pires R, Brolin H, Delannoy M, Atkinson GC. FlaGs and webFlaGs: discovering novel biology through the analysis of gene neighbourhood conservation. Bioinformatics. 2021;37: 1312–1314. doi:10.1093/bioinformatics/btaa788

5. Kos VN, Deraspe M, McLaughlin RE, Whiteaker JD, Roy PH, Alm RA, et al. The resistome of Pseudomonas aeruginosa in relationship to phenotypic susceptibility. Antimicrob Agents Chemother. 2015;59: 427–36. doi:10.1128/AAC.03954-14

6. Trouillon J, Imbert L, Villard A-M, Vernet T, Attrée I, Elsen S. Determination of the two-component systems regulatory network reveals core and accessory regulations across Pseudomonas aeruginosa lineages. Nucleic Acids Res. 2021;49: 11476–11490. doi:10.1093/nar/gkab928

7. Roy PH, Tetu SG, Larouche A, Elbourne L, Tremblay S, Ren Q, et al. Complete Genome Sequence of the Multiresistant Taxonomic Outlier Pseudomonas aeruginosa PA7. PLOS ONE. 2010;5: e8842. doi:10.1371/journal.pone.0008842

8. Holloway BW. Genetic recombination in Pseudomonas aeruginosa. J Gen Microbiol. 1955;13: 572–581. doi:10.1099/00221287-13-3-572

9. Rahme LG, Stevens EJ, Wolfort SF, Shao J, Tompkins RG, Ausubel FM. Common virulence factors for bacterial pathogenicity in plants and animals. Science. 1995;268: 1899–1902.

10. Reboud E, Elsen S, Bouillot S, Golovkine G, Basso P, Jeannot K, et al. Phenotype and toxicity of the recently discovered *exlA* -positive *Pseudomonas aeruginosa* strains collected worldwide: Virulence of exlA+ strains. Environ Microbiol. 2016;18: 3425–3439. doi:10.1111/1462-2920.13262

11. Kessler B, de Lorenzo V, Timmis KN. A general system to integratelacZ fusions into the chromosomes of gram-negative eubacteria: regulation of thePm promoter of theTOL plasmid studied with all controlling elements in monocopy. Mol Gen Genet MGG. 1992;233: 293–301. doi:10.1007/BF00587591

12. Rietsch A, Vallet-Gely I, Dove SL, Mekalanos JJ. ExsE, a secreted regulator of type III secretion genes in Pseudomonas aeruginosa. Proc Natl Acad Sci. 2005;102: 8006–8011. doi:10.1073/pnas.0503005102
